# Integrated multi-omics reveals anaplerotic rewiring in methylmalonyl-CoA mutase deficiency

**DOI:** 10.1038/s42255-022-00720-8

**Published:** 2023-01-26

**Authors:** Patrick Forny, Ximena Bonilla, David Lamparter, Wenguang Shao, Tanja Plessl, Caroline Frei, Anna Bingisser, Sandra Goetze, Audrey van Drogen, Keith Harshman, Patrick G. A. Pedrioli, Cedric Howald, Martin Poms, Florian Traversi, Céline Bürer, Sarah Cherkaoui, Raphael J. Morscher, Luke Simmons, Merima Forny, Ioannis Xenarios, Ruedi Aebersold, Nicola Zamboni, Gunnar Rätsch, Emmanouil T. Dermitzakis, Bernd Wollscheid, Matthias R. Baumgartner, D. Sean Froese

**Affiliations:** 1 Division of Metabolism and Children’s Research Center, University Children’s Hospital Zurich, University of Zurich, Zurich, Switzerland; 2 Biomedical Informatics, Department of Computer Science, Swiss Federal Institute of Technology/ETH Zürich, Zurich, Switzerland; 3 Health 2030 Genome Center, Geneva, Switzerland; 4 PHRT Swiss Multi-Omics Center, smoc.ethz.ch, Zurich, Switzerland; 5 Institute of Translational Medicine, Department of Health Science and Technology, Swiss Federal Institute of Technology/ETH Zürich, Zurich, Switzerland; 6 Swiss Institute of Bioinformatics, Lausanne, Switzerland; 7 Department of Biology, Institute of Molecular Systems Biology, Swiss Federal Institute of Technology/ETH Zürich, Zurich, Switzerland; 8 Division of Clinical Chemistry, University Children’s Hospital Zurich, University of Zurich, Zurich, Switzerland; 9 Division of Oncology and Children’s Research Center, University Children’s Hospital Zurich, University of Zurich, Zurich, Switzerland; 10 Department of Pediatric and Adolescent Oncology, Gustave Roussy Cancer Center, Université Paris-Saclay, Villejuif, France; 11 Division of Child Neurology, University Children’s Hospital Zurich, University of Zurich, Zurich, Switzerland; 12 Agora Center, Lausanne, Switzerland; 13 Medical Informatics Unit, University Hospital Zurich, Zurich, Switzerland; 14 AI Center, ETH Zurich, Zurich, Switzerland; 15 Department of Genetic Medicine and Development, University of Geneva Medical School, Geneva, Switzerland

**Keywords:** Metabolism, Mechanisms of disease, Paediatric research, Transcriptomics

## Abstract

Methylmalonic aciduria (MMA) is an inborn error of metabolism with multiple monogenic causes and a poorly understood pathogenesis, leading to the absence of effective causal treatments. Here we employ multi-layered omics profiling combined with biochemical and clinical features of individuals with MMA to reveal a molecular diagnosis for 177 out of 210 (84%) cases, the majority (148) of whom display pathogenic variants in methylmalonyl-CoA mutase (*MMUT*). Stratification of these data layers by disease severity shows dysregulation of the tricarboxylic acid cycle and its replenishment (anaplerosis) by glutamine. The relevance of these disturbances is evidenced by multi-organ metabolomics of a hemizygous *Mmut* mouse model as well as through identification of physical interactions between MMUT and glutamine anaplerotic enzymes. Using stable-isotope tracing, we find that treatment with dimethyl-oxoglutarate restores deficient tricarboxylic acid cycling. Our work highlights glutamine anaplerosis as a potential therapeutic intervention point in MMA.

## Abstract (web summary)

The authors combine multi-layered omics with clinical and biochemical features from individuals affected with methylmalonic aciduria, a rare inherited disease affecting succinyl-CoA synthesis, revealing that anaplerotic rewiring is a targetable feature.

## Main

Inborn errors of metabolism (IEMs), first described by Archibald Garrod^1^, are inherited diseases resulting from the inadequate function of metabolic proteins. IEMs represent a group of nearly 1,500 diseases with a combined incidence of approximately 1:800 births. They present a clinically and genetically heterogeneous picture making them inherently difficult to diagnose^2,3^. Beyond their diagnostic challenges, the pathomechanisms of many IEMs are not well understood; hence most IEMs lack rationalized treatment approaches^4^.

Methylmalonic aciduria (MMA) is a prototypic IEM that may be caused by defects in approximately 20 genes^5^. Classical isolated MMA is an autosomal recessive disorder caused by pathogenic variants in the genes *MMUT*, *MMAA* and *MMAB*, with a prevalence of approximately 1:90,000 births^6^. The gene products of *MMAA* and *MMAB* convert intracellular vitamin B_12_ (cobalamin, Cbl) into its cofactor form (adenosylcobalamin, AdoCbl), which is used by methylmalonyl-CoA mutase (MMUT) for the breakdown of methylmalonyl-CoA to succinyl-CoA as part of propionate catabolism. Dysfunction of any of these proteins leads to the accumulation of the eponymous metabolite methylmalonic acid and others. Clinically, individuals with classical isolated MMA frequently present with failure to thrive and acute life-threatening episodes in the neonatal period, involving vomiting and impaired neurological function (comatose state and metabolic stroke) accompanied by biochemical disturbances (metabolic acidosis and hyperammonemia)^7^. Surviving patients with MMA are affected by long-term complications that mainly include neurological abnormalities (movement disorder and intellectual impairment), kidney failure and anemia/neutropenia^7^. Even though the (dys)function of MMUT has been studied extensively^8–11^, the main metabolic disturbances and pathomechanisms in MMA remain an open question and curative treatment options are not available.

Technological advances in genomics and mass spectrometry, leveraging datasets of whole molecule classes (omics), have recently led to a paradigm shift in their use as diagnostic tools. For example, single-layer WGS has achieved diagnostic rates of 30–50% in rare disease cohorts^12–14^, while dual-layer combination with RNA sequencing (RNA-seq) can improve this by 10–35%^15–18^. Despite these advances, a substantial number of patients with IEM remain undiagnosed and disease course prediction remains poor, mainly due to a lack of pathomechanistic understanding and often unclear genotype–phenotype relationships.

Multi-layered omics data have the potential to not only increase diagnosis rates of IEMs but also to uncover mechanistic insights into disease pathophysiology^19^, thus potentially indicating new therapeutic targets. Such a combinatorial approach is key to moving beyond the traditional ‘one gene, one disease’ view of these disorders, which fails to explain phenotypic heterogeneity based on genetic variation only; however, the simultaneous application of multi-omics technologies for this purpose has not been rigorously tested and their true utility, bottlenecks and knowledge gaps remain unknown.

By combining whole-genome sequencing (WGS), whole transcriptome sequencing (RNA-seq) and proteotyping information (data-independent acquisition mass spectrometry (DIA–MS)) with phenotypic features, we identified disease-causative and pathogenic features in a cohort of MMA-affected individuals. We revealed underlying damaging variants and differentially expressed transcripts and proteins directly related to anaplerosis of the tricarboxylic acid (TCA) cycle. Moreover, follow-up studies utilizing untargeted metabolomics and [U-^13^C]glutamine tracing revealed a depletion of TCA cycle anaplerosis in line with the identified dysregulation of glutamate dehydrogenase and oxoglutarate dehydrogenase enzymes, which we found to physically interact with a complex of proteins, including MMUT. Beyond unveiling these metabolic disturbances in MMUT deficiency, our findings enable a better biological understanding of TCA cycle anaplerosis. Furthermore, the anaplerotic TCA cycle insufficiency in MMA may be a potential therapeutic intervention point, as demonstrated by boosting TCA cycle intermediate pools and reducing MMA-specific toxic metabolites by dimethyl-oxoglutarate treatment.

## Results

### Monogenic disease variant detection through multi-omics

**Fig. 1:**
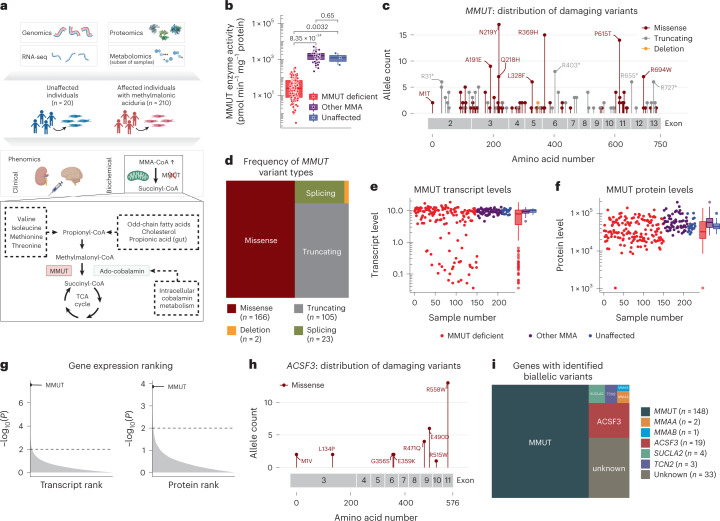
Multi-faceted omics view enabled a molecular diagnosis in 84% of individuals. **a**, Study overview with a depiction of the propionate pathway, including its precursors and the pathways catalyzed by MMUT. **b**, MMUT enzyme activity per study sub-cohort (MMUT-deficient, *n* = 150; other MMA, *n* = 60; unaffected, *n* = 3); *P* values were calculated by Wilcoxon rank test, two-sided. **c**, Lollipop plot of all pathogenic variants found on the *MMUT* gene. **d**, Proportions of variant types as identified on the *MMUT* gene. **e**,**f**, Transcript and protein levels of MMUT by study sub-cohorts (number of samples for the transcript and protein plot, respectively: MMUT-deficient, *n* = 143/150; other MMA, *n* = 59/60; unaffected, *n* = 19/20). **g**, Gene ranks according to *P* values as calculated by gene-wise Welch’s *t*-test (two-sided) in the proteomics and transcriptomics data. **h**, Lollipop plot of pathogenic variants identified in *ACSF3*. **i**, Proportions of affected genes identified in the whole cohort. *P* values were calculated by the Wilcoxon rank test. Boxplot elements represent center line, median; box limits, upper and lower quartiles; whiskers, 1.5× interquartile range; and points, outliers in **e** and **f**. Dots in **b**,**e**,**f** represent biologically independent samples. Source data

**Extended Data Fig. 1:**
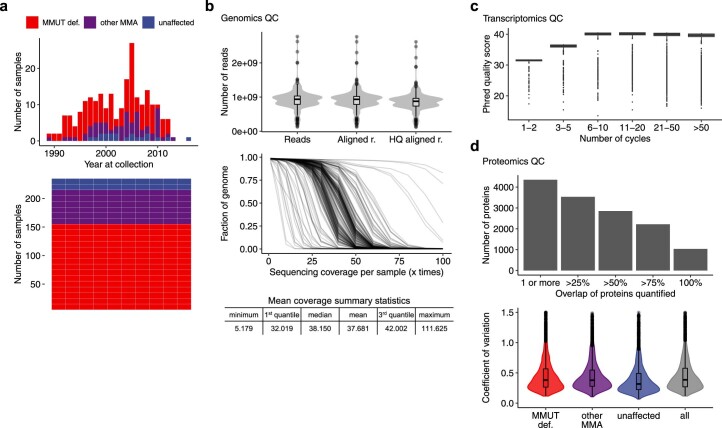
Historic context of sample collection and quality control measurements of multi-omics data. **a**, Histogram of fibroblast samples binned into their year at collection and waffle chart illustrating the different sample groups indicated by the color code. **b**, Violin plots illustrating average number of read per sample (n = 229). HQ, high quality. Line plot (one line per sample) indicating genome coverage as quantitatively summarized in the below table. **c**, Boxplots indicating Phred quality scores at different number of cycles. Each sample (n = 221) underwent 75 cycles that were binned (see *x* axis) to display the Phred scores per bin (bin 1−2, 1206 scores; 3−5, 1809; 6−10, 3015; 11−20, 6030; 21−50, 18090; > 50, 15075). **d**, Proteomics quality control illustrated by the number of detected proteins in relation to the percentage of overlapping proteins (top panel). Using a threshold of > 50% overlapping proteins, the variation coefficients of n = 2850 proteins are displayed for each sample group. Boxplot elements represent center line, median; box limits, upper and lower quartiles; whiskers, 1.5x interquartile range; points, outliers. Violin plots depict the distribution of the data using vertical density curves.

**Extended Data Fig. 2:**
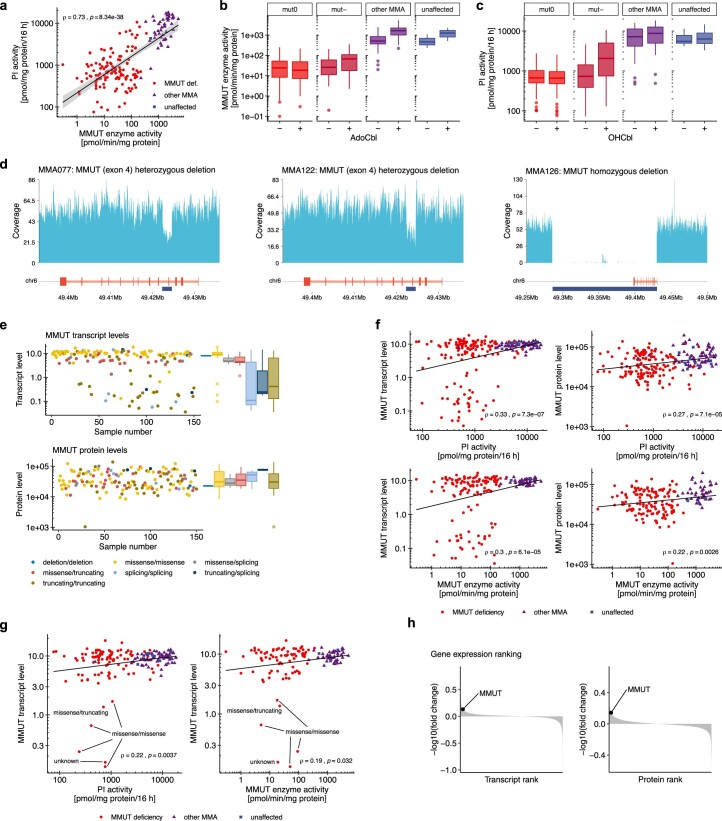
Biochemical assessment of MMUT activity and propionate incorporation activity supports diagnosis of affected individuals. **a**, Scatter plot of maximal, that is supplemented with adenosylcobalamin (AdoCbl) or hydroxocobalamin (OHCbl), activity of the MMUT enzyme and the propionate incorporation assay. **b**, Boxplots of MMUT enzyme activity with and without AdoCbl supplementation measured in biologically independent fibroblast samples (*mut*^0^, n = 119; *mut*^-^, n = 29; other MMA, n = 46; unaffected, n = 3). **c**, Boxplots of propionate incorporation activity with and without OHCbl supplementation measured in biologically independent fibroblast samples (*mut*^0^, n = 120; *mut*^-^, n = 30; other MMA, n = 60; unaffected, n = 9). **d**, Copy number variants illustrated by read counts of specific locations of the *MMUT* gene for three specific samples. **e**, Scatter plots of MMUT transcript and protein levels of the MMUT-deficient samples. Samples are indicated by dots and are grouped according to the underlying bi-allelic genetic variation type of the *MMUT* gene (Number of samples for the transcript and protein plot, respectively: deletion/deletion, n = 1/1; missense/missense, n = 63/65; missense/splicing, n = 6/6; missense/truncating, n = 26/30; splicing/splicing, n = 6/6; truncating/splicing, n = 3/3; truncating/truncating, n = 36/37). **f**, Regression plots of MMUT transcript and protein levels versus MMUT enzyme and propionate incorporation activity. **g**, Same as f but excluding samples with truncating/truncating, splicing/splicing and truncating/splicing *MMUT* allele combinations. **h**, Fold change of all transcripts and proteins, respectively, when comparing the MMUT-deficient group versus the rest of the samples. Gene names are ranked according to the negative base 10 logarithm of the fold change. All linear regressions are calculated according to the Pearson method, *P* values two-sided; bands indicate 95% confidence level interval. Boxplot elements represent center line, median; box limits, upper and lower quartiles; whiskers, 1.5x interquartile range; points, outliers.

To extend the understanding of MMA from the causative genomic lesions to the affected biochemical processes, we performed high-quality WGS, RNA-seq and DIA–MS-based proteotyping on fibroblasts taken from 230 individuals (210 affected by MMA and 20 unaffected), representing a mainly European cohort collected over 25 years (Fig. 1a and Extended Data Fig. 1). Biochemical assay of propionate incorporation (PI)^20^ and MMUT enzyme activity^8^ was strongly correlated across all samples (rho = 0.73, *P* < 0.0001) (Extended Data Fig. 2a). Fibroblasts from 150 individuals with MMA had reduced MMUT activity (MMUT-deficient), including 123 that did not increase upon cofactor supplementation (Extended Data Fig. 2b,c), whereas those of 60 individuals had MMUT activity similar to controls (other MMA) (Fig. 1b).

In the MMUT-deficient samples, we searched the WGS dataset for disease-causing variations in the *MMUT* gene and identified the molecular cause of disease in 148 out of 150 individuals (Supplementary Table 1). Pathogenic variants constituted 165 missense alleles, 105 truncating alleles, 21 splicing alleles, 2 alleles with in-frame deletions and 3 alleles containing copy-number variants (Fig. 1c,d and Extended Data Fig. 2d), of which 41 variants were new (Supplementary Table 1). RNA-seq identified reduced *MMUT* RNA expression in cells from MMUT-deficient individuals compared to the other groups (Fig. 1e). Individuals with strongly reduced RNA expression were enriched for splicing and/or truncating variants, consistent with nonsense-mediated decay (Extended Data Fig. 2e). DIA–MS-based proteome measurements revealed reduced MMUT protein levels in MMUT-deficient primary fibroblasts (Fig. 1f), which were distributed across all variant types (Extended Data Fig. 2e). Consistent with its disease-causing role, MMUT RNA and protein levels were positively and significantly associated with PI and MMUT activity (Extended Data Fig. 2f), without truncating or splicing alleles driving this correlation (Extended Data Fig. 2g). MMUT represented the most significantly dysregulated RNA and protein of MMUT-deficient samples when compared to all other samples (Fig. 1g and Extended Data Fig. 2h).

**Extended Data Fig. 3:**
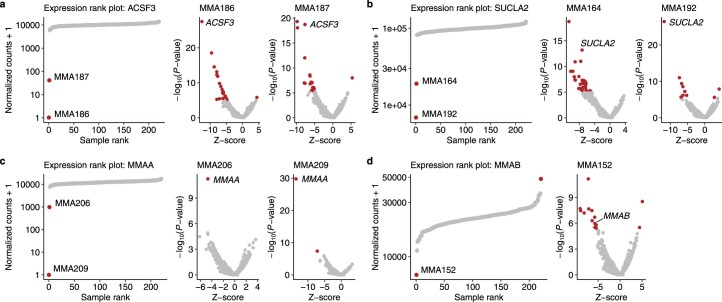
Expression outlier analysis reveals causative genes in specific disease samples. Expression rank plots for **a**, *ACSF3*, **b**, *SUCLA2*, **c**, *MMAA* and **d**, *MMAB* and Z-score volcano plots for specific samples, applying the OUTRIDER R package.

In the remaining 60 samples, we identified bi-allelic disease-causing variants for 22 individuals in genes other than *MMUT*: *ACSF3* (17 individuals) (Fig. 1h), *TCN2* (3 individuals), *SUCLA2* (1 individual) and *MMAB* (1 individual) according to the ACMG classification (Supplementary Table 1). By searching RNA-seq for aberrantly expressed genes using OUTRIDER^21^ (Extended Data Fig. 3), we identified two individuals with very low *ACSF3* expression; two with aberrant *SUCLA2* expression, in whom we confirmed predicted splicing and copy-number variants at the genomic level (Supplementary Table 1); two with very low *MMAA* expression, confirmed by complementation analysis; and one with low *MMAB* transcript, also confirmed by complementation analysis. Therefore, we identified a molecular cause for 29 out of 60 remaining individuals (48%), including 18 pathogenic mutations, of which 10 were new (Supplementary Table 1). In summary, we found a diagnosis for 177 out of 210 (84%) affected individuals (Fig. 1i), including 150 with deficiency of MMUT and 19 with damaging variants in *ACSF3*, accounting for the largest cohort of ACSF3 deficiency.

### Phenotypic description and association with disease severity

**Fig. 2:**
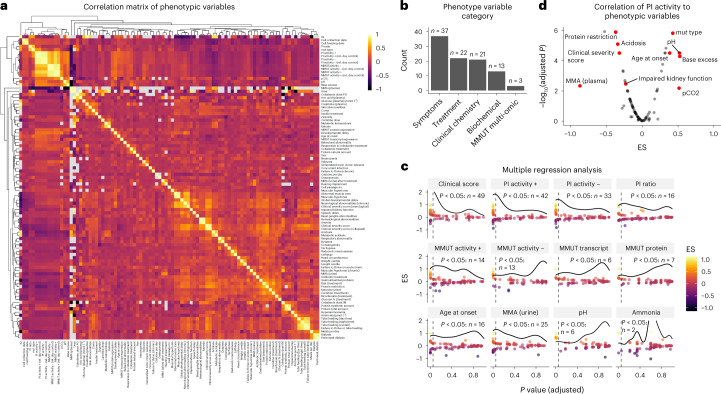
Phenomics analysis reveals two main surrogate markers of disease severity (CSS and PI+ activity). **a**, Correlation matrix of all continuous numeric and discrete phenotype variables. **b**, Number of phenotypic traits according to five phenotype subcategories. **c**, Panel of selected phenotypic traits and their overall strength of representing the entirety of the phenomics dataset (here termed disease severity) as assessed by linear modeling after log transformation. Each point represents the result of linear regression against one other phenotypic variable with the effect size (ES) on the *y* axis and the resulting Benjamini–Hochberg adjusted *P* value (two-sided) on the *x* axis. The horizontal curved line indicates the density of data points as distributed along the *x* axis. The vertical dashed line indicates the threshold of significance (*P* < 0.05). **d**, Linear regression results of the PI+ activity variable compared to the rest of the phenotypic variables; *P* values calculated as in **c**. Source data

We expected that the genetic underpinnings identified above would only partly predict the clinical and biochemical phenotypes of affected individuals as other genetic (for example, the combination of pathogenic alleles and gene regulation) or non-genetic factors (for example, protein–protein interactions) might influence the manifestation of MMA disease. We therefore aimed at establishing a quantitative assessment of disease severity by converting the catalog of mostly semantic phenotypic traits into key numeric variables. In total we collected 105 phenotypic variables. Following exclusion of nine unspecific and/or interdependent features (Methods; Cohort Selection), we generated a correlation matrix of phenotypic variables (*n* = 96), spanning clinical symptoms at presentation and during disease course (*n* = 37), clinical treatments and therapeutic response (*n* = 22), clinical chemistry of blood or tissues including metabolite measurements (*n* = 21) and in vitro biochemical parameters (*n* = 13), revealed a cluster of features (MMUT activity, PI) that showed strong correlation across many variables (Fig. 2a,b).

**Extended Data Fig. 4:**
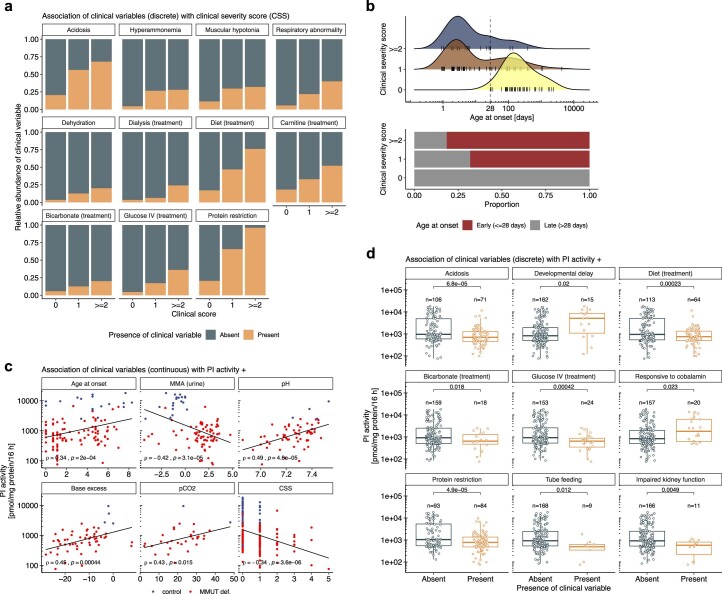
The clinical severity score and propionate incorporation activity are associated with several phenotypic traits. **a**, Proportional bar plots of the presence of absence of clinical parameters in relation to the clinical severity score. **b**, Age at onset in relation to the clinical severity score. **c**, Linear regression of various relationships of propionate incorporation activity to continuous phenotypic variables (Pearson method, two-sided, pairwise comparisons). **d**, Comparison of propionate incorporation activity with discrete phenotype variables (*P* values by *t*-test, two-sided, pairwise comparisons; number of samples per boxplot are indicated above the boxplot). Boxplot elements represent center line, median; box limits, upper and lower quartiles; whiskers, 1.5x interquartile range. Each dot represents a sample/patient.

As the identified few variables are strongly associated with many clinical features, we postulated that most disease characteristics might be well predicted by one or a select few variables. As a proof of principle, we established a clinical severity score (CSS), which incorporated the outcome of five typical clinical features^7^ (composition in Methods), whereby a score of 0 represented the absence of these typical MMA features, a score of 1 indicated mild MMA and a score of 2 or higher (maximum 5) indicated moderate to severe MMA disease. Comparison of the CSS against all phenotypic parameters demonstrated a significant correlation with 49 individual variables (Fig. 2c), including many classical phenotypic symptoms of MMAuria, such as acidosis, hyperammonemia and muscular hypotonia, as well as the requirement for dietary and pharmacological interventions (Extended Data Fig. 4a). Notably, the CSS also inversely correlated with age of onset (Extended Data Fig. 4b), a parameter that on its own has been used as an indication of clinical severity^22^.

Multiple correlation analysis identified PI in the presence of hydroxocobalamin (PI+) to significantly correlate with 42 phenotypic features, the most of any individual continuous variable (Fig. 2c). This contrasts, for example, to age at onset, which was significantly correlated with only 16 parameters (Fig. 2c). Closer inspection revealed PI+ to be inversely correlated with disease severity, including significant positive correlation with, for example, pH or age at disease onset and negative correlation with, for example, methylmalonic acid concentration in plasma, presence of clinical interventions such as protein restriction and the CSS (Fig. 2d and Extended Data Fig. 4c,d). Of note, these relationships were preserved when all collected features were included (Supplementary Fig. 1). Therefore, in line with its validity as a diagnostic test for MMA^20^, the PI+ variable was used as an approximation of clinical disease severity in this study.

### Disruption of the TCA cycle and associated pathways

To identify disease-associated expression alterations of genes, proteins and pathways, we attempted a global assessment of transcript and protein expression integrated with the quantitative phenotype variables identified above. As patients with TCN2, SUCLA2 and ACSF3 deficiency lack most of the typical signs and symptoms of classical MMA, we compared MMUT-deficient with all non-MMUT-deficient samples (control).

**Fig. 3:**
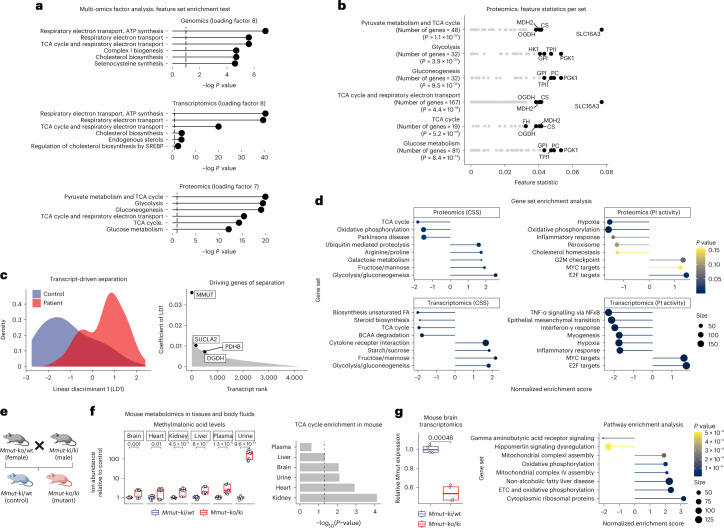
Untargeted integration of omics data layers highlights the TCA cycle and associated pathways as well as oxidative phosphorylation gene sets to be dysregulated in MMA. **a**, Gene set enrichment test using the multi-omics factor analysis tool (MOFA). Benjamini–Hochberg adjusted *P* values (two-sided) are shown. **b**, Detailed feature statistics of the top enriched gene sets following MOFA in the proteomics data; *P* values calculated as in **a**. **c**, Linear discriminant model (split to assign training and test data, 0.5) of transcripts separates MMUT-deficient from control driven by *MMUT* and other genes related to the TCA cycle. **d**, Gene set enrichment analysis based on ES ranking derived from differential expression analysis (also Fig. 4b); *P* values were calculated with the fgsea R package. **e**, Breeding scheme of *Mmut*-deficient mice. **f**, Untargeted metabolomics in mouse tissues and body fluids, depicting boxplots for methylmalonic acid and metabolite set enrichment analysis based on the complete metabolomics dataset. Significantly changing ions between mutant and control conditions were identified using a two-sample *t*-test. Pathway analysis was performed using an annotated ion list ranked by *P* value significance. Pathway enrichments were calculated using KEGG metabolic pathway definitions and a hypergeometric test. **g**, RNA-seq on mouse brain tissue. Boxplots of the relative *Mmut* transcript abundance and gene set enrichment analysis following DESeq2 analysis; dot size represents the number of genes per set. *P* values in **f** and **g** (boxplot) are calculated by Wilcoxon rank test, two-sided. Boxplot elements represent center line, median; box limits, upper and lower quartiles; and whiskers, 1.5× interquartile range. Source data

Investigation of transcripts and proteins using differential correlation patterns (Pearson correlation method), dimensionality reduction via principal-component analysis and DESeq2 did not immediately yield a clear grouping of the data nor obvious expression pattern differences between the groups (Supplementary Fig. 2); however, multi-omics factor analysis^23^, integrating both genetic data layers and proteotyping data, identified mitochondrial metabolic pathways and, in particular, the electron transport chain and the TCA cycle to be enriched in MMUT-deficient samples (Fig. 3a). In more detail, the proteins SLC16A3, CS, MDH2 and OGDH were found to be the main drivers of this particular factor’s variance in the proteotyping data within the TCA-associated gene sets (Fig. 3b). Linear discriminant analysis of genes shared between transcriptomics and proteotyping indicated MMUT as the strongest and SUCLA2, OGDH and PDHB to be top drivers of separation between MMUT-deficient and control samples (Fig. 3c). Further, gene set enrichment analysis utilizing sample stratification by disease severity, both by CSS and PI+, also identified oxidative phosphorylation and the TCA cycle as over-represented pathways in the proteomics (CSS and PI+) and transcriptomics (CSS) datasets (Fig. 3d).

These changes were consistent with findings in a hemizygous mouse model of MMA^24^ (Fig. 3e). Untargeted metabolomics of brain, heart, kidney, liver, plasma and urine confirmed elevated levels of the eponymous metabolite methylmalonic acid in mutant animals, whereas pathway enrichment analysis pointed to dysregulated TCA cycle pathways in all tissues and urine (Fig. 3f). Transcriptomics of brain tissue further confirmed the expected 50% reduction in *Mmut* transcript of mutant mice, along with enrichment of electron transport chain and oxidative phosphorylation pathways (Fig. 3g, Supplementary Fig. 3).

### MMUT deficiency leads to alterations in proximal TCA enzymes

**Fig. 4:**
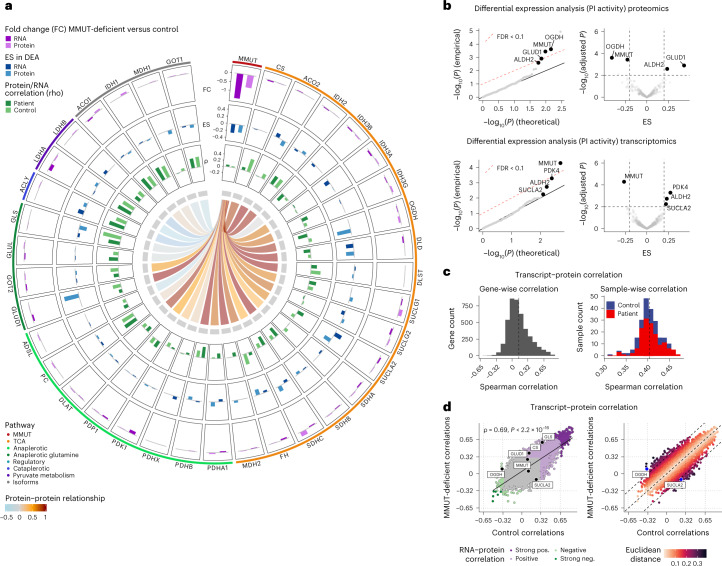
Transcript–protein and protein–protein correlation analyses reveal coordinated relationships between MMUT and TCA genes and proteins but not their isoforms. **a**, Circos plot depicting raw fold changes (FC) of transcripts and proteins, effects sizes (ES) derived from differential expression analysis, transcript–protein correlations (rho) and correlative relationships of the MMUT protein to TCA proteins and their corresponding isoforms. **b**, Q-Q and volcano plots illustrate the results of the differential expression analysis based on a linear mixed modeling approach applied to the proteomics and transcriptomics data, restricted to enzymes (or their encoding genes) localized in the mitochondria; calculated *P* values were two-sided. **c**, Histograms of Spearman correlations across 4,318 transcript–protein pairs (left) and 221 samples (right). **d**, Scatter plot of Spearman correlations in MMUT-deficient against control. Euclidean distance from the diagonal is calculated based on the formula (MMUT-deficient correlation−control correlation)/sqrt(2); calculated *P* values were two-sided.

As both data-driven and phenotypically stratified analyses indicated TCA and associated pathways to be disrupted in disease, we performed a concerted investigation of the TCA cycle enzymes, including those that metabolize anaplerotic (replenishing TCA cycle intermediates) and cataplerotic (removing TCA cycle intermediates) reactions, from which we had both RNA and protein information. As controls, we included isoforms of TCA enzymes that are not involved in these pathways (Fig. 4a). Direct comparison of RNA and protein expression between MMUT-deficient and control cells revealed MMUT to be significantly dysregulated (Fig. 4a outer band).

Differential expression analysis, performed using a linear mixed modeling approach^25^, identified the genes with the strongest effect size and significance to be enriched for mitochondrial localization, as listed in MitoCarta 3.0 (ref. ^26^) (Supplementary Fig. 4). Closer examination (Fig. 4a,b middle band), identified MMUT to be significantly downregulated in disease at both the RNA and protein level, whereas ALDH2, which catalyzes the interchange between methylmalonate and methylmalonate semialdehyde, was upregulated in both. A further upregulated transcript was *PDK4* (Fig. 4b), which is responsible for the phosphorylation and, as a consequence, inactivation of the pyruvate dehydrogenase complex; however, the proteins with the overall largest effect size were OGDH (downregulated in disease) and GLUD1 (upregulated in disease), both enzymes involved in the anaplerosis of glutamine (Fig. 4b). In line with our findings, proteomics studies in human MMA livers by others have equally identified upregulated GLUD1 and ALDH2 (ref. ^27^) and targeted analysis of liver-derived isolated mitochondria from a murine MMA model showed decreased OGDH protein levels and enzyme activity^28^.

**Extended Data Fig. 5:**
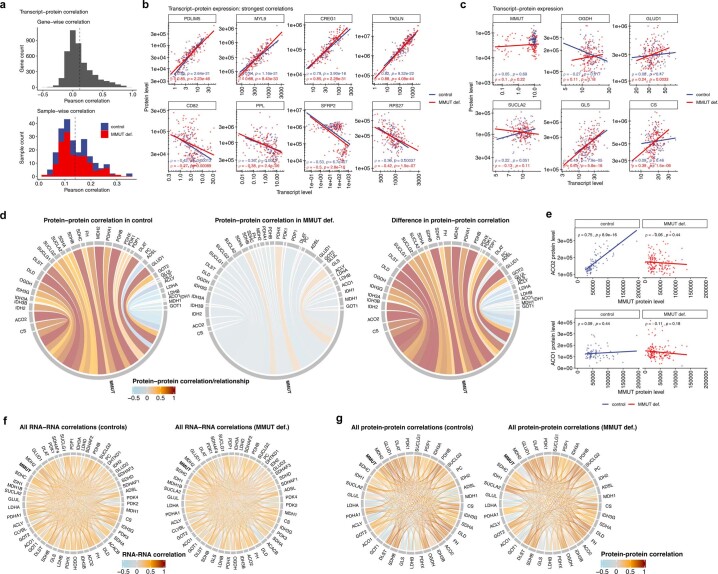
Transcript–protein and protein–protein correlation analysis illustrates coordinated regulation of MMUT with most TCA transcripts and proteins. **a**, Histograms of Pearson correlations across 4318 transcript–protein pairs (top) and 221 samples (bottom). **b**, Scatter plot of the strongest positive and negative transcript–protein correlations (ranked by average of Pearson correlation coefficient in the MMUT-deficient and control group). **c**, Transcript–protein Pearson correlation plots of selected TCA cycle related genes. **d**, Spearman correlation of the MMUT protein versus a selection of TCA cycle and related proteins and their isoforms illustrated in a chord plot for control (left), MMUT-deficient samples (middle) and the difference of the two former plots (right); thickness of the links indicates nominal value of the correlation coefficient. **e**, Scatter plot of MMUT protein versus both isoforms of aconitase (ACO1 and ACO2) with linear regression by Pearson correlation. **f**, Chord plot illustrating all correlative relationships of TCA cycle and related transcripts or proteins (**g**); thickness of the links indicates nominal value of the Spearman correlation coefficient. All *P* values calculated two-sided.

Examination of RNA–protein expression correlation in all samples revealed a limited Spearman correlation of 0.14 at the gene level (4,318 transcript–protein pairs) and 0.40 at the sample level (Fig. 4c and Extended Data Fig. 5a), similar to findings by others^29^. RNA–protein correlation in MMUT-deficient cells compared to controls revealed that, while 1,158 pairs (26.8%) correlated significantly (*P* < 0.05) in both genotypes (Fig. 4d all colored points and Extended Data Fig. 5b) in accordance with previous studies^30^, the correlation of some genes segregated depending on the genotype (MMUT-deficient versus control) (Fig. 4d). In particular, OGDH, GLUD1, CS and GLS showed higher RNA–protein correlation in MMUT-deficient samples than controls, whereas SUCLA2 had reduced RNA–protein correlation (Fig. 4a,d and Extended Data Fig. 5c). *OGDH* and *SUCLA2* were among the genes with the strongest genotype-dependent RNA–protein correlation changes (Fig. 4d). Notably, we found poor RNA–protein correlation for MMUT in both control and MMUT-deficient cells (Fig. 4a,d and Extended Data Fig. 5c).

Finally, MMUT protein levels positively correlated with protein levels of many TCA and anaplerotic enzymes in control but not in MMUT-deficient cells, whereas there was little or no protein expression correlation between MMUT and non-TCA protein isoforms in either genotype (Fig. 4a center and Extended Data Fig. 5d). Such a relationship is exemplified by MMUT:ACO2 and MMUT:ACO1 (Fig. 4a center and Extended Data Fig. 5e) and indicates that MMUT may be part of a so far unknown interaction network with these mitochondrial TCA cycle and anaplerotic enzymes^31^. Examination of pairwise correlation between all proteins and transcripts (Extended Data Fig. 5f,g) in these pathways suggests that the TCA cycle and anaplerotic enzymes have a positive correlation with each other, which is not altered in MMUT deficiency unless MMUT is included in the comparison. Overall, the above findings suggest that disruption of MMUT RNA and protein expression drives regulatory changes in certain TCA and anaplerotic enzymes.

### Metabolomics highlights rewiring of TCA cycle anaplerosis

**Fig. 5:**
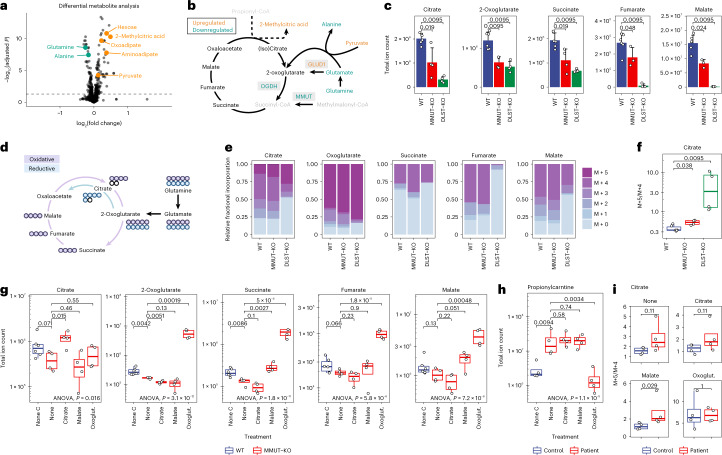
Polar metabolomics and glutamine tracing studies in CRISPR/Cas9 KO 293T cells and primary patient fibroblasts highlight differential glutamine anaplerosis. **a**, Volcano plot depicting differentially expressed metabolites. Highlighted are those particularly relevant to this study. **b**, Schematic depiction of the TCA cycle and relevant anaplerotic reactions. The color code indicates dysregulations at the metabolite and protein levels; gray metabolites were not detected. **c**, Pool sizes of metabolites in control and CRISPR/Cas9 KO 293T cells (error bars represent s.d., centered around the mean). **d**, Schematic representation of labeling of TCA cycle and associated metabolites derived from labeled glutamine via anaplerosis. Circles represent carbon atoms. **e**, Relative abundance of isotopologs of TCA cycle metabolites after glutamine labeling. **f**, Ratios of M + 5 and M + 4 citrate isotopologs. **g**, Total pool sizes of TCA cycle metabolites under different treatment conditions. Oxoglut., dimethyl-oxoglutarate. **h**, Levels of propionylcarnitine in primary patient fibroblasts under treatment. **i**, Ratios of M + 5 and M + 4 citrate isotopologs under treatment in primary fibroblasts; *P* values were calculated by two-sided Wilcoxon rank test. Boxplot elements represent center line, median; box limits, upper and lower quartiles; and whiskers, 1.5× interquartile range. For experiments in 293T cells (**c**,**e**–**g**), *n* = 3 biologically independent samples (WT), *n* = 2 (MMUT-KO), *n* = 2 (DLST-KO) over two independent experiments were measured. For experiments in patient fibroblasts (**h**,**i**), *n* = 4 biologically independent samples per group were measured. Source data

**Extended Data Fig. 6:**
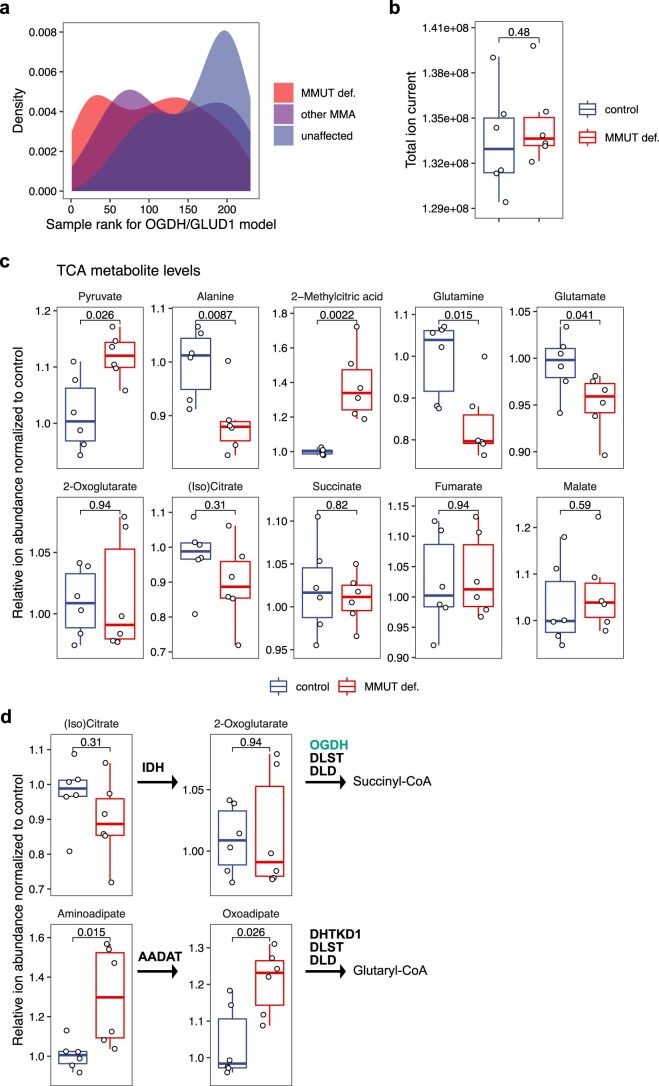
Metabolomics investigation of a subset of patient cell lines. **a**, Density plot illustrating a model for OGDH and GLUD1 with all fibroblast samples ranked according to three sample groups. **b**, Boxplots of total ion current assessed in the two experimental groups ‘control’ and ‘MMUT-deficient’ primary fibroblasts (n = 6 in each group); technical replicates are collapsed to represent one dot per cell line. **c**, TCA metabolites as measured by untargeted polar metabolomics; n = 6 biological replicates. **d**, Levels of metabolites involved in the two enzymatic steps catalyzed by two oxoacid dehydrogenase complexes (OGDC and OADC) and their proximal reactions; OGDH protein in green indicates its downregulation as detected in the proteotyping dataset; n = 6 biological replicates. Boxplot elements represent center line, median; box limits, upper and lower quartiles; whiskers, 1.5x interquartile range. All *P* values are calculated by Wilcoxon rank test, two-sided.

To examine the functional consequences of the above RNA and protein expression alterations, we performed untargeted metabolomic analysis on a set of six MMUT-deficient fibroblasts, derived from patients, and six control primary fibroblasts, derived from unaffected individuals (selection criteria in Extended Data Fig. 6a and Methods). While the total ion current was comparable in the MMUT-deficient and control samples (Extended Data Fig. 6b), we found decreased glutamine and alanine as well as increased hexoses, methylcitrate, oxoadipate, aminoadipate and pyruvate among the most significantly changed metabolites (Fig. 5a). No strong pool-size changes of TCA cycle intermediates were observed in these cells (Extended Data Fig. 6c); however, many of the altered metabolites represent anaplerotic precursors. Changes in anaplerotic metabolites are consistent with observed changes in RNA and protein expression in the matching model systems, indicating disruption of TCA cycle anaplerosis in MMUT deficiency (Fig. 5b). They also suggest a potential knock-on effect to adjacent pathways, as illustrated by the increased oxoadipate and aminoadipate (Fig. 5a). These are upstream metabolites of the 2-oxoadipate dehydrogenase complex, which shares its E2 (DLST) and E3 (DLD) components with the 2-oxoglutarate dehydrogenase complex (Extended Data Fig. 6d), potentially indicative of a preference for 2-oxoglutarate over 2-oxoadipate metabolism in MMUT-deficient cells.

**Extended Data Fig. 7:**
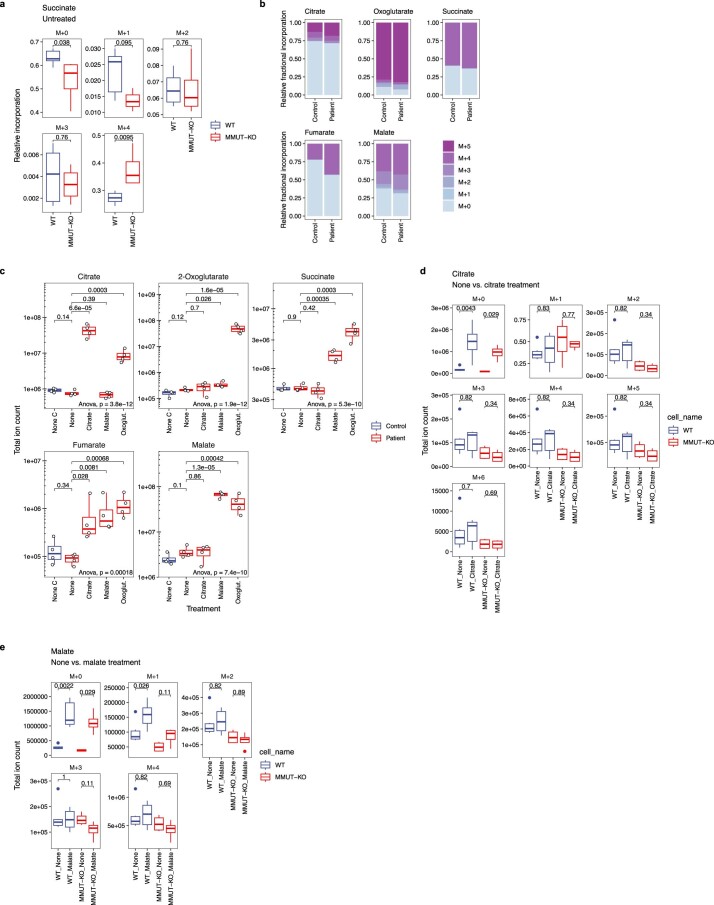
Treatment of primary patient/control fibroblasts and 293T cells. **a**, Relative incorporation into different isotopologues of succinate without treatment of 293T cells. **b**, Relative abundance of isotopologues of TCA cycle metabolites after glutamine labeling. **c**, Total pool sizes of TCA cycle metabolites under different treatment conditions (Oxoglut., dimethyl-oxoglutarate) in primary fibroblasts; n = 4 biologically independent samples per group were measured. **d**, Total ion count for citrate and malate (**e**) is displayed for different isotopologues under citrate and malate treatment, respectively. For each boxplot representing results from 293 T cells (**c**, **e**, **f**, **g**), n = 3 biologically independent samples (WT), n = 2 (MMUT-KO), n = 2 (DLST-KO) over 2 independent experiments were measured. For experiments in patient fibroblasts (h, i), n = 4 biologically independent samples per group were measured; *P* values calculated by Wilcoxon rank test, two-sided. Boxplot elements represent center line, median; box limits, upper and lower quartiles; whiskers, 1.5x interquartile range; dots in a, d, and e are outliers, in c individual samples.

To study anaplerotic alterations in-depth, we performed targeted metabolomics in 293T cells with a wild-type (WT), *MMUT* knockout (KO) or *DLST*-KO genetic background, as validated by Western blotting analysis and enzyme activity measurements (Supplementary Fig. 5). *DLST*-KO cells were used as an additional control to mimic the reduction of OGDH protein in MMUT deficiency. In *MMUT*-KO cells, we found markedly reduced TCA cycle intermediates, indicating an overall reduced TCA metabolite pool, whereas KO of *DLST* led to virtual absence of most TCA cycle metabolites (Fig. 5c). In addition, we found reduced pool sizes of glutamine and glutamate (Supplementary Fig. 6a) comparable to the patient fibroblasts (Extended Data Fig. 6c). These results point to an adjusted reliance on the glutamine anaplerotic pathway in disease; a hypothesis we tested further by assessing relative carbon incorporation derived from [U-^13^C]glutamine into TCA cycle and associated intermediates (Fig. 5d). To identify differential labeling patterns, we studied the isotope distribution based on the relative incorporation of glutamine-derived carbons (Supplementary Fig. 6b) into the TCA cycle. In this experiment, immediate anaplerotic glutamine catabolism results in M + 5 (glutamate and oxoglutarate) and M + 4 (succinate, fumarate, malate and citrate) compounds, whereas oxidative cycles of the TCA cycle will incorporate mostly unlabeled carbon (for example, from glucose and methylmalonyl-CoA) and dilute glutamine-derived ^13^C. In *MMUT*-KO conditions, we found a tendency for increased proportional fractions of M + 5 oxoglutarate and M + 4 isotopologs of all studied TCA cycle metabolites, with reduced proportional fractions of M + 0 for each (Fig. 5e), as exemplified by succinate (Extended Data Fig. 7a). This implies that cells with impaired MMUT have an increased use of glutamine as an anaplerotic source. Moreover, consistent with reduced OGDH activity, there was a relative preference for the reductive TCA cycle pathway, as indicated by an increased M + 5/M + 4 ratio for citrate (Fig. 5d)^32,33^ in MMUT deficiency (Fig. 5f). Applying the same labeling technique in primary patient and control fibroblasts replicated the observed TCA cycle rewiring (Extended Data Fig. 7b).

**Extended Data Fig. 8:**
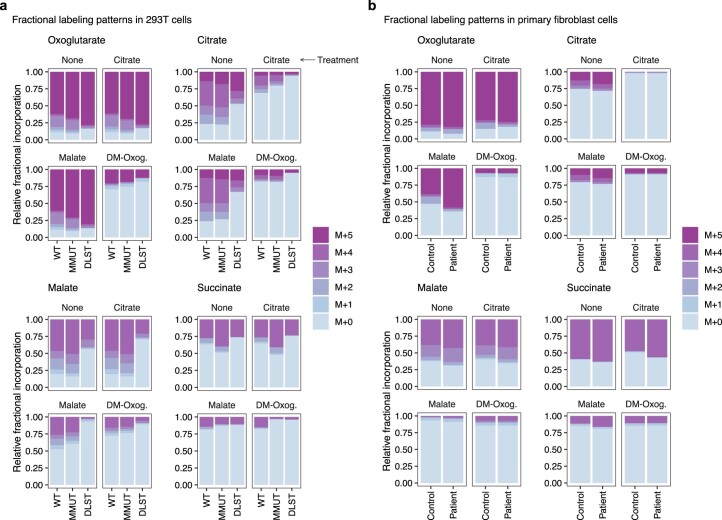
Fractional labeling pattern derived from glutamine in 293T and primary fibroblast cells upon treatment. Cells (**a**, 293T. **b**, primary patient and control fibroblasts) treated with the compounds indicated above the plots (DM-Oxog., dimethyl-oxoglutarate).

To test whether direct supplementation of core TCA-related metabolites can correct the reduced pools of TCA cycle intermediates, we re-performed metabolomics studies either without intervention or following supplementation with citrate, malate or dimethyl-oxoglutarate in 293T cells and fibroblasts (Fig. 5 and Extended Data Fig. 7c). Dimethyl-oxoglutarate is a membrane-permeable alternative to 2-oxoglutarate, previously used in a model of OXPHOS dysfunction^32^. Supplementation with citrate and malate increased pools of the respective intracellular metabolites but did not have a broad impact on other TCA intermediates (Fig. 5g and Extended Data Fig. 7c). As we did not separate mitochondrial and cytosolic pools, the majority of supplemented citrate may have remained cytosolic, consistent with the largely enhanced total peak area of M + 0 citrate but no change to the amount of labeled citrate (Extended Data Fig. 7d). Likewise, the slightly broader effect of malate may reflect its partial entrance into the mitochondria, although labeled malate pools were also relatively unchanged following supplementation (Extended Data Fig. 7e). Dimethyl-oxoglutarate, in contrast, in addition to increasing oxoglutarate pools, increased succinate, malate and fumarate in 293T cells (Fig. 5g) as well as citrate in primary patient fibroblasts (Extended Data Fig. 7c). It can be speculated that this is due to its high penetrance into the mitochondria by masking the negative charges with methyl groups. These patterns were reinforced by investigation of [U-^13^C]glutamine-derived labeling patterns, whereby the addition of citrate and malate ablated the contribution of glutamine to their respective pools, but did not strongly affect the anaplerotic synthesis of other intermediates, whereas dimethyl-oxoglutarate reduced the anaplerotic contribution of glutamine to all intermediates detected (Extended Data Fig. 8a,b). Two other observations make dimethyl-oxoglutarate an interesting treatment compound: in patient fibroblasts, dimethyl-oxoglutarate strongly reduced pools of propionylcarnitine, an MMA disease biomarker and derivative of the toxic metabolite propionyl-CoA (Fig. 5h), and it led to a tendency to decrease the M + 5/M + 4 ratio for citrate in MMUT deficiency (Fig. 5i), indicating a potential throttling and re-balancing of reductive TCA cycling under this treatment.

### MMUT physically interacts with other anaplerotic enzymes

**Fig. 6:**
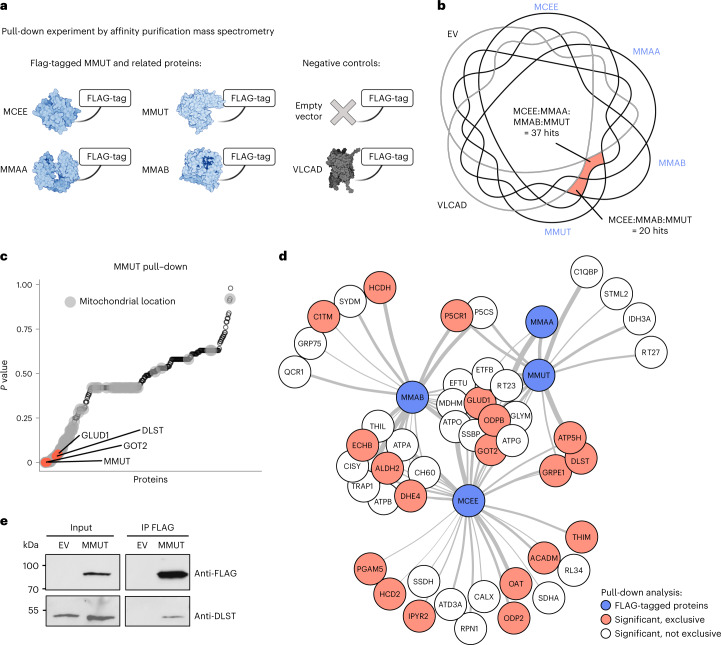
MMUT interacts physically with GLUD1, DLST and GOT2 as demonstrated by FLAG-tag pull-down. **a**, Outline of experimental and control groups indicating which protein was used with a FLAG-tag in a cross-linking affinity purification experiment coupled to subsequent analysis of the pull-down samples by mass spectrometry. **b**, Venn diagram of proteins pulled down by the different FLAG-tagged proteins. **c**, ANOVA *P* values (two-sided) of all proteins pulled down by MMUT. Gray dots indicate proteins with mitochondrial location, according to UniProt. **d**, Interaction network of significantly enriched proteins (ANOVA *P* value <0.05, two-sided). Thicker connector indicates lower *P* value. Blue, proteins with FLAG-tags used in pull-down; red, significantly pulled-down proteins as indicated in red in **b** that do not share any peptides with the negative controls; no color, significantly enriched proteins, but not exclusively pulled down by FLAG-tagged proteins. **e**, Western blot of IP of FLAG-tagged MMUT probing for DLST. Data are representative of three independent experiments. Source data

**Extended Data Fig. 9:**
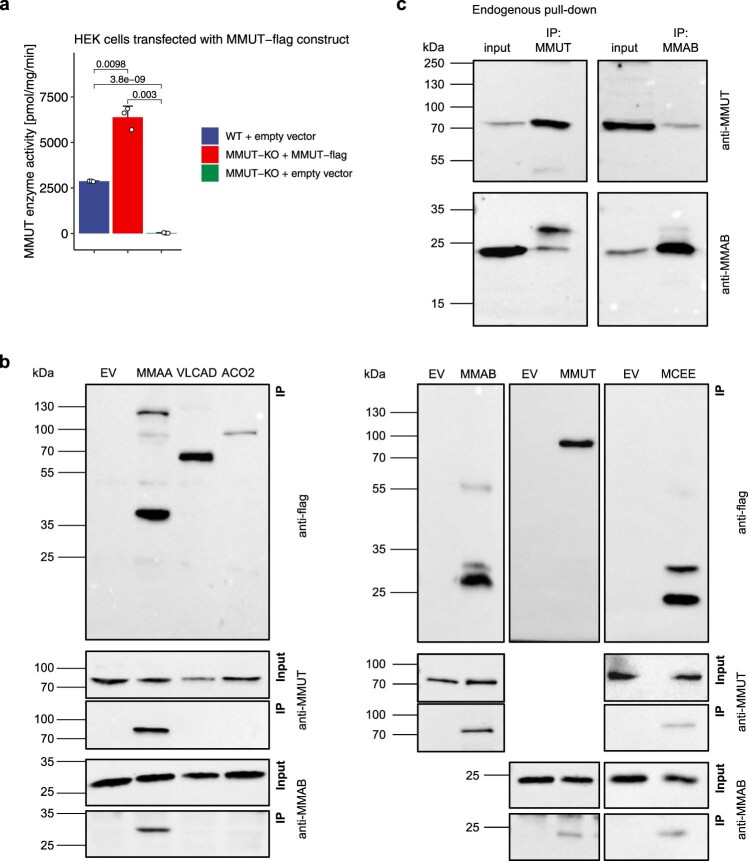
MMUT-flag is enzymatically active and pulls down other propionate pathway proteins using immunoprecipitation. **a**, MMUT enzyme activity using a radio-labeled substrate in 293T cells (backgrounds (WT or MMUT-KO) and transfected constructs are indicated in the color key; data points indicate means of n = 3 independent experiments; error bars indicate SD, centered around the mean; *P* values calculated by *t*-test, two-sided. **b**, Western blots of over-expressed flag-tagged MMUT, MMAB, MMAA, and MCEE, but not VLCAD, ACO2, and empty vector (EV). **c**, Detection of endogenous MMUT and MMAB following cross-linking immunoprecipitation. Panels shown are representative of at least n = 3 independent experiments. Source data

The above-observed adjustments in glutamine anaplerosis have an unclear regulatory etiology. On the basis of the strong protein expression correlation between MMUT and proximal TCA cycle and anaplerotic enzymes (Fig. 4a and Extended Data Fig. 5d), we hypothesized that these proteins might be part of a shared metabolon complex, potentially facilitating regulation of TCA cycle anaplerosis of glutamine by protein–protein interactions^31^. To gain insights into this potential physical relationship, we took advantage of the over-expression of C-terminally FLAG-tagged versions of functional MMUT (Extended Data Fig. 9a), three pathway members involved in MMUT cofactor (MMAA and MMAB) and substrate (MCEE) synthesis expected to participate in any multi-protein complex containing MMUT and three negative controls (empty vector (EV), VLCAD and ACO2) in 293T cells (Fig. 6a). Using cross-linking immunoprecipitation (IP) followed by immunoblotting, we found that IP of over-expressed MMUT, MMAB, MMAA and MCEE enabled detection of endogenous MMUT and MMAB (Extended Data Fig. 9b), a result confirmed by reciprocal detection of endogenous MMAB and MMUT (Extended Data Fig. 9c), indicating that these pathway members were indeed part of a complex. In contrast, IP following expression of EV, VLCAD and ACO2 did not result in the detection of endogenous MMUT and MMAB (Extended Data Fig. 9b), demonstrating that the above-detected interactions are specific.

**Extended Data Fig. 10:**
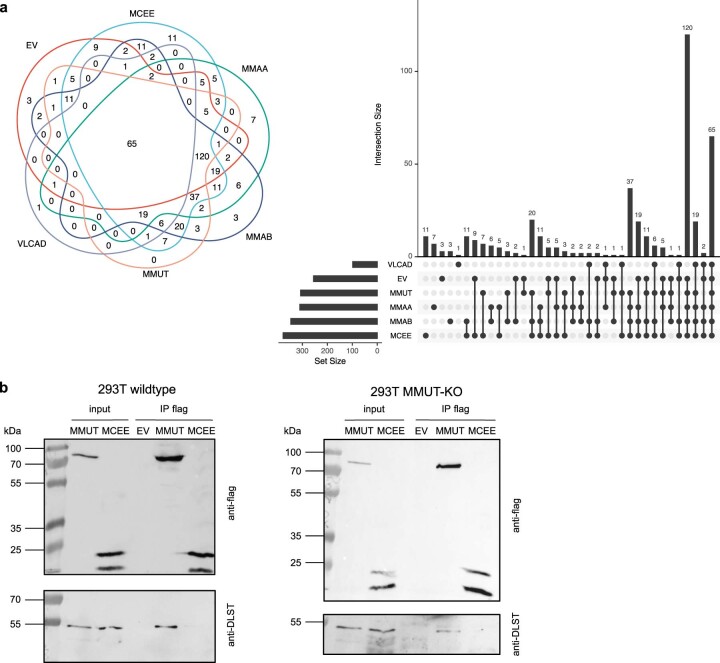
Quantitative pull-down results following affinity purification mass spectrometry and confirmation of DLST pull-down by MMUT-flag by immunoprecipitation. **a**, Numbers indicate count of proteins per intersection following affinity purification mass spectrometry; Venn regions are labeled with the names of flag-tag bait proteins; UpSet plot illustrates intersection and set sizes. **b**, Immunoprecipitation of flag-tagged MMUT and MCEE probing for DLST in 293T WT and MMUT-KO cell lines. Panels shown are representative of at least n = 3 independent experiments. Source data

To examine a potential complex with TCA cycle and anaplerotic enzymes, we performed IP coupled to mass spectrometry of the four MMUT pathway proteins (MMUT, MMAA, MMAB and MCEE) and two negative controls (EV and VLCAD). At 1.0% false discovery rate (FDR) using two peptides minimum at 95% threshold, each of these ‘bait’ proteins pulled down a total of 100–350 different ‘prey’ proteins over three biological replicates (Extended Data Fig. 10a). Within this intersection, we identified 37 prey proteins pulled down by MCEE, MMAA, MMAB and MMUT but not by EV or VLCAD in any replicate, including MMUT, OGDH, DLST and GOT2, as well as 20 proteins pulled down by MCEE, MMAB and MMUT but not EV or VLCAD including MMAB and GLUD1 (Fig. 6b). Analysis of variance (ANOVA) of the biological triplicates comparing MMUT with EV and VLCAD identified 22 proteins to be significantly enriched (nominal *P* value <0.05) in the MMUT sample (Fig. 6c and Supplementary Table 2). All proteins were designated by UniProt to have mitochondrial localization and included GLUD1, GOT2 and DLST (Fig. 6c). ANOVA comparing the intersection of proteins confidently pulled down by at least three of MMUT, MMAA, MMAB and MCEE but neither of EV or VLCAD identified 11 interacting proteins, including GLUD1 and GOT2; whereas the intersection of two of MMUT, MMAA, MMAB and MCEE but neither negative control identified 13 interacting proteins, including DLST (Fig. 6d and Supplementary Table 2). Finally, complex formation between MMUT and DLST was additionally confirmed by immunoblotting (Fig. 6e and Extended Data Fig. 10b). These data indicate that MMUT is part of a complex of proximal metabolic enzymes, including GLUD1 and the oxoglutarate dehydrogenase complex component DLST and suggests that disruption of these interactions may underlie their altered regulation in disease.

## Discussion

In this study, we used an integrated multi-modal approach to diagnose and uncover pathomechanisms of the IEM like MMA. Unique to this investigation was the relatively large set of patient samples and corresponding phenotypes available for such a rare genetic disease and the ability to coordinate aliquots from the same samples to generate data at three molecular layers. The results of our study will encourage future endeavors to use our approach in any setting of an inborn monogenic disease. Moving forward, the datasets derived from our study can be further exploited, for example, by applying network contextualization tools^34^, integrating multi-omics and flux modeling^35^ and reconstructing genome-scale metabolic networks^36^, continuing to refine the pipeline of a multi-modal study of IEMs.

Our findings reinforce the value of comprehensive and complementary datasets to increase diagnostic yield and the understanding of the pathophysiological underpinnings of disease. Our multi-modal profiling allowed the identification of causative genetic variation in 84% of the cohort, including causative factors in the samples without MMUT deficiency. We were able to widen the set of genes beyond the classical MMA genes *MMUT*, *MMAA* and *MMAB*. For example, the identification of *ACSF3* damaging variants in our cohort is particularly notable as they have recently been linked to combined malonic and MMA^37^. The phenotype of patients with combined malonic and MMA was indistinguishable from the remainder of patients with MMA with normal MMUT activity, highlighting the fact that IEMs present with widely overlapping phenotypes and that they should be studied with large gene panels or with WGS approaches to avoid biases toward known genes and to augment the chances of diagnosis.

While the ability of clinical phenotypic information to predict a molecular diagnosis was limited, phenotypic variables, both clinical and biochemical, enabled sample stratification by disease severity and consequently identification of multi-level alterations of metabolic genes/proteins that were not apparent following examination of single omics layers. Such a move away from ‘data silos’ into true integrative and mechanism-based, multi-layered analysis remains challenging, as it requires new analytical and statistical methods to combine these disparate datasets^38^. In this capacity, multi-omics factor analysis^23^ highlighted the disruption to transcripts and proteins of the TCA cycle and related pathways, a finding verified by the correlation with phenotypic data utilizing both PI activity and a CSS. Following multi-modal integration, we performed metabolomics in select patient cells and further complemented the data with glutamine tracing and protein–protein interaction studies in a second cell model. In summary, these experiments showed decreased TCA metabolite pools and an increased glutamine-derived anaplerosis. Similar investigations in the MMA-related disorder propionic aciduria showed limited flux derived from ^13^C-labeled α-oxoglutarate^39^. In addition, we found previously unidentified MMUT-interaction candidates, among which DLST (OGDH complex component) and GLUD1 are directly involved in the anaplerotic glutamine pathway. It is of note that such a tailored set of follow-up experimental approaches (orthogonal to multi-omics data) is invaluable for molecular assessment of potential targets and the validation of their biological significance.

Our results highlight the importance of the loss of methylmalonyl-CoA as an anaplerotic source and indicate a relevant reduction of TCA cycle intermediates in MMA. We show that anaplerotic insufficiency is a relevant pathomechanism of MMA and addressed this phenomenon as a therapeutic target by treating both our cellular models with TCA cycle intermediates. Such anaplerotic stimulating approaches have precedent in IEMs, including the application of triheptanoin in long-chain fatty acid oxidation disorders^40^ or citrate treatment of patients suffering from the MMA-related disorder propionic aciduria^41^. Our findings now show that dimethyl-oxoglutarate, a membrane-permeable alternative to 2-oxoglutarate, previously used in a model of OXPHOS dysfunction^32^, may represent a more promising therapeutic strategy. Studies to further delineate the efficacy of such approaches in preclinical and clinical models will be important for the ongoing development of new treatments for MMA and IEMs in general.

Here, we studied a unique cohort of a rare IEM. The cohort is remarkable with regard to the number of included patients with rare diseases, amount of phenotypic information collected and availability of primary fibroblast cell lines for every individual in the cohort; however, future efforts are required to include every individual (including an equal number of controls) in an unbiased way to avoid collider bias and to collect phenotypic data in a complete, standardized and longitudinal manner (for example, via rare disease registries); aims that were not possible in our multinational, multi-decade cohort. Further, corroboration of our findings of TCA cycle rewiring in MMA will be required in orthogonal models, including in vivo studies, to assess their applicability in a therapeutic setting.

## Methods

### Cohort and patient-derived fibroblast samples

Primary fibroblast samples and corresponding disease-related information, including clinical and diagnostic data, were collected from 1989 to 2015. The information obtained and the use of fibroblasts remains under the ethics approval granted by the Ethics Committee of the Canton of Zurich (KEK-2014-0211, amendment: PB_2020-00053). Upon collection, primary fibroblasts were cultured using Dulbecco’s modified Eagle’s medium (DMEM; Gibco, Life Technologies) with 10% fetal bovine serum (Gibco) and antibiotics (GE Healthcare) and either used immediately or exchanged with 90% fetal bovine serum and 10% dimethyl sulfoxide and stored in cryovials under liquid nitrogen. A frozen aliquot of each primary fibroblast cell culture was sent for WGS, RNA-seq and DIA-MS analysis (Fig. 1a). RNA-seq and DIA–MS were always performed from matched aliquots.

### Cohort selection

Patient samples were referred to our center initially for enzymatic or genetic diagnostic purposes. For this study, we selected affected individuals (*n* = 210) based on the presence of methylmalonic acid in urine or plasma. Patient samples were accompanied by a questionnaire filled by the referring physician (Supplementary Document 1) containing data on the patients’ clinical and biochemical presentation. Phenotype data are provided in the Source Data for Fig. 2. For the analyses shown in Fig. 2, nine phenotypic variables (hypothermia, hyperventilation, irritability, somnolence, vomiting, dehydration, feeding difficulties, responsive to acute treatment and estimated glomerular filtration rate) were excluded due to their nonspecific nature, whereas the analyses in Supplementary Fig. 1 included complete phenotype information. Control samples were obtained from healthy individuals or donors without a biochemical defect whose diagnosis excluded MMA.

### Clinical disease severity score

The clinical disease severity score was based on five typical clinical signs/symptoms of MMA^7^, including age at disease onset, as well as the presence of neurological abnormalities, kidney impairment, hematological abnormalities and failure to thrive. Each patient was assigned a score from 0–5, indicating increasing disease severity (Source Data for Fig. 2).

### Biochemical activity assays

PI into acid-precipitable material of primary fibroblasts was assessed according to a protocol described previously^42^ with modifications as described^20^. MMUT enzyme activity assay was performed in fibroblast crude cell lysates as originally described^43,44^ using recent modifications^8^. MMUT enzyme activity in HEK cells was measured using the same protocol but without radiolabeled substrate (instead only 1 mM of methylmalonyl-CoA was used, Sigma M1762) and final succinate determination was performed by HPLC separation and electrospray ionization (ESI) tandem mass spectrometry (MS/MS) detection (SCIEX TripleQuad 5500 LC–MS/MS System).

### WGS

Genomic DNA was isolated using QIAmp DNA Mini kit reagents (QIAGEN) following the protocol provided by the supplier. WGS libraries were prepared with TruSeq DNA PCR-free library reagents (Illumina) using 1 μg of genomic DNA following the protocol provided by the supplier. The genomic DNA libraries were quantified using the KAPA Library Quantification Complete kit (Roche) according to the protocol supplied with the reagents. The quantified libraries were sequenced on the NovaSeq 6000 sequencer (Illumina) using a 150-nucleotide paired-end-run configuration following the protocol provided by the supplier.

### RNA-seq

Total RNA was isolated using the Rneasy Plus Mini kit (QIAGEN). RNA-seq libraries were prepared using the TruSeq Stranded mRNA-seq reagents (Illumina) using 200 ng of total RNA following the protocol provided by the supplier. The quality of the total RNA and the RNA-seq libraries was assessed on Fragment Analyzer (Agilent). The libraries were sequenced on Illumina HiSeq 4000 using the 75-nucleotide paired-end-run configuration following the protocol provided by the supplier.

### Sample preparation for mass spectrometry proteotyping measurements

Samples were processed in blocks of eight, taking into consideration a balance between disease types and control samples. All other factors within a block were randomized. A total of 230 samples were processed in three batches. For sample processing, aliquots of primary fibroblast (~1 × 10^6^ cells per vial) were washed twice in ice-cold PBS (Gibco), resuspended in lysis buffer (Preomics) at a ratio of 1:1 (vol pellet/vol lysis buffer) and incubated at 95 °C for 10 min. Samples were sonicated in a vial tweeter (Hielscher Ultrasound Technology) at 4 °C for three cycles with an amplitude 100%, power 80% during 30 s. Then, 100 μg of protein lysate were further processed with the iST kit (Preomics). The purified peptides were resuspended in LCLoad buffer containing iRT peptides (Biognosys) at a concentration of 1 μg μl^−1^.

### Spectral library generation

For spectral library generation, three times 24 samples (3 × 8 sample blocks) were pooled. Pooled sample batches were digested as described above. Then, 100 μg of purified peptides were fractionated on a C18 column (YMC-Triart, C18, 3 μm, 250 × 0.5 mm internal diameter) according to pH on an Agilent HPLC 1260 system with a stepped 61-min gradient ranging from 95 % buffer A (20 mM ammonium formate acid/H_2_O) to 85% buffer B (20 mM ammonium formate/90% ACN). Overall, 48 fractions were collected per sample and subsequently pooled to 24 fractions. Samples were resuspended in 5% ACN/0.1% FA and analyzed on a Q-Exactive HF-X mass spectrometer (Thermo Fisher Scientific) in DDA mode. The same nLC 1200 configuration and mobile phase gradient elution conditions as for DIA were applied.

Full MS survey scans were acquired at a resolution of 60,000 with automatic gain control (AGC) target of 3 × 10^6^ and a maximum injection time of 45 ms over a scan range of m/z 375–1,500. A data-dependent top-12 method was used for HCD MS/MS with a normalized collision energy of 28 at a resolution of 15,000 and a fixed first mass of m/z 100. Precursor ions were isolated in a 1.4-Th window and accumulated to reach an AGC target value of 1 × 10^5^ with a maximum injection time of 22 ms. Precursor ions with a charge state of 1 and 6 as well as isotopes were excluded for fragmentation. Dynamic exclusion was set to 15 s.

DDA raw files were processed with Proteome Discoverer (v.2.2) using a human UniProt database (release 201804) together with iRT peptides (Biognosys) and common contaminants. The processing workflow consisted of SequestHT^45^ and Amanda^46^ nodes coupled with Percolator^47^. The following search parameters were used for protein identification: (1) a peptide mass tolerance of 10 ppm; (2) an MS/MS mass tolerance of 0.02 Da; (3) fully tryptic peptide search with up to two missed cleavages were allowed; and (4) carbamidomethylation of cysteine was set as fixed modification, methionine oxidation and protein N-terminal acetylation were set as variable modifications. Percolator was set at max deltaCN of 0.05, with target FDR strict of 0.01 and target FDR relaxed of 0.05. The spectral library from Proteome Discoverer was imported into Spectronaut v.12 (Biognosys) using standard parameters with 0.01 peptide spectrum match FDR.

### DIA–MS setup and data analysis

For DIA analysis, samples were measured on a Q-Exactive HF mass spectrometer (Thermo Fisher Scientific). Mobile phase A consisted of HPLC-grade water with 0.1% (v/v) formic acid and mobile phase B consisted of HPLC-grade ACN with 20% (v/v) HPLC-grade water and 0.1% (v/v) formic acid. Peptide separation was carried out on an ES806, 2 µm, 100 Å, 150 µm internal diameter × 150 mm, C18 EASY-Spray column (Thermo Fisher Scientific) at a temperature of 50 °C. For LC–MS/MS analyses, 2 μg each sample were loaded onto the column via an Easy-nLC 1200 system (Thermo Fisher Scientific). Samples were loaded at 4 µl min^−1^ with 100% mobile phase A for 5 min. Peptide elution was performed using the following gradient (1) 2% to 8% mobile phase B in 4 min; (2) 8% to 32% mobile phase B in 49 min; (3) 32% to 60 % mobile phase B in 1 min; and (4) ramp to 98% mobile phase B in 1 min at 2 µl min^−1^.

For DIA acquisition on a Q-Exactive HF mass spectrometer, we applied a DIA method published elsewhere^48^. In short, we performed an MS1 scan over a mass range of m/z 400–1210 at a resolution of 120,000 with an AGC target value of 3 × 10^6^ and with a maximum injection time of 50 ms. For MS/MS scans, the resolution was at 30,000 with an AGC target value of 1 × 10^6^ and with ‘Auto’ maximum injection time. Precursor ions were isolated within a 15-Th window and fragmented by HCD with normalized collision energy 28. A total of 54 MS/MS scan windows were defined, interspersed every 18 scans with an MS1 scan.

DIA data analysis was performed in Spectronaut v.12 (Biognosys) using standard parameters. For identification, a Q value cutoff of 0.01 was applied on the precursor as well as on the protein level. The MS1 area was selected for quantification. Quantification parameters were set to mean peptide quantity for major group quantity, the top three peptides were selected for protein quantity calculation. Data filtering was set to *Q* value sparse, with no imputation. Cross-run normalization was set to local. The protein report for downstream analysis contained information report about PG.ProteinAccessions, PG.ProteinDescriptions, PG.ProteinNames. PG.Qvalue and PG.Quantity.

### Quality assessment of WGS, RNA-seq and DIA–MS data

Overall quality assurance tests revealed a mean of high-quality aligned genomic reads of 8.7 × 10^8^ at a median genomic coverage of > 38-fold (Extended Data Fig. 1b). A median of 3.74 million single-nucleotide variations were called using the Genome Analysis Toolkit^49^ and DeepVariant^50^. RNA-seq data showed a median Phred score of > 36.3 at three and more cycles (Extended Data Fig. 1c), while proteomics data showed a high reproducibility with 2,218 proteins detected in at least 75% of samples (Extended Data Fig. 1d). For 9 of the 230 samples RNA extraction yielded insufficient nucleic acid to proceed with transcriptome sequencing; hence, these datasets were excluded from all further analysis (transcriptomics data of sample IDs 22, 54, 59, 78, 89, 109, 123, 207 and 221).

### Selection of primary fibroblasts for polar metabolomics

To select cell lines for metabolomics, we opted for a balanced design with ten MMUT-deficient cell lines and ten control lines. MMUT-deficient lines were picked to show over-expression of GLUD1 and under-expression of OGDH, whereas the control lines were chosen to show the reverse pattern. We fitted a mixed-effects model with PI+ as a response, two fixed effects for GLUD1 and OGDH expression and a random effect with the same covariance structure as the proteomics data after column and row normalization. From the MMUT-deficient and control cell lines, we chose ten with the lowest predicted value of PI+ and ten with the highest predicted value, respectively. The top-ten ranked MMUT-deficient (**MMA014**, MMA092, **MMA042**, **MMA067**, MMA093, **MMA104**, **MMA013**, MMA030, MMA138 and **MMA036**) and the last-ten ranked control primary fibroblasts (MMA219, MMA221, MMA227, **MMA222**, **MMA213**, **MMA230**, MMA226, **MMA228**, **MMA225** and **MMA215**) were selected and cultured as described above. Six primary fibroblast lines (in bold above) met growth criteria and were selected for the polar metabolomics experiment.

### Fibroblast sample preparation for polar metabolomics

A total of 100,000 cells per well were seeded in a six-well plate and grown for 48 h. Medium was removed and cells were washed twice with 150 mM ammonium hydrogen carbonate (NH_4_HCO_3_) at pH 7.4. The whole plate was flash-frozen in liquid nitrogen for 20 s and then stored at −80 °C. Metabolites were extracted by putting the plate on dry ice and adding cold (−20 °C) 40:40:20 acetonitrile:methanol:water and incubated at −20 °C for 10 min. Supernatant was collected and a second volume of 40:40:20 acetonitrile:methanol:water was added and incubated at −20 °C for 10 min. Plates were put on dry ice and cells were scraped mechanically and collected. Collection tubes were centrifuged at 15,000*g* for 2 min at 4 °C, supernatants were collected and stored at −20 °C before metabolomics analysis.

### Polar metabolomics in patient-derived fibroblasts

Untargeted metabolite profiling was performed using flow injection analysis on an Agilent 6550 QTOF instrument (Agilent) using negative ionization, 4 GHz high-resolution acquisition and scanning in MS1 mode between m/z 50–1,000 at 1.4 Hz^51^. The solvent was 60:40 isopropanol:water supplemented with 1 mM NH_4_F at pH 9.0, as well as 10 nM hexakis(1H, 1H, 3H-tetrafluoropropoxy)phosphazine and 80 nM taurochloric acid for online mass calibration. The seven batches were analyzed sequentially. Within each batch, the injection sequence was randomized. Data were acquired in profile mode, centroided and analyzed with MatLab (Mathworks). Missing values were filled by recursion in the raw data. Upon identification of consensus centroids across all samples, ions were putatively annotated by accurate mass and isotopic patterns. Starting from the HMDB v.4.0 database, we generated a list of expected ions, including deprotonated, fluorinated and all major adducts found under these conditions. All formulas matching the measured mass within a mass tolerance of 0.001 Da were enumerated. As this method does not employ chromatographic separation or in-depth MS2 characterization, it is not possible to distinguish between compounds with the identical molecular formula. The confidence of annotation reflects level 4 but, in practice, in the case of intermediates of primary metabolism, it is higher because they are the most abundant metabolites in cells. The resulting data matrix included 1,809 ions that could be matched to deprotonated metabolites listed in HMDB. All m/z peaks that remained unmatched or were associated with adducts or heavy isotopomers were discarded.

### Mouse care and handling

The study was approved under license no. 202/2014 from the Cantonal Veterinary Office Zurich. Generation of the *Mmut*-p.Met698Lys variant model and crossing with a *Mmut*-ko/wt model was conducted as previously described^24^. These mice, B6.129S1-Mmut<tm1Pai>×B6-Mmut<tm1.1Mrb> were generated on a C57BL/6J background. Mice were housed in single-ventilated cages with a 12-h light–dark cycle and an artificial light of approximately 40 Lux in the cage. The animals were kept under controlled humidity (45–55%) and temperature (21 ± 1 °C) and housed in a barrier-protected specific-pathogen-free unit. Mice had ad libitum access to sterilized drinking water and to pelleted and extruded mouse diet containing 18.5% protein and 4.5% fat (Kliba-Nafag, 3436).

### Collecting of mouse tissues

Urine was collected in the morning after one night in a metabolic cage. The sediment was removed and the supernatant was flash-frozen in liquid nitrogen. Tissue samples were collected from mice aged 58–63 d. Animals were anesthetized by sevoflurane. Portal blood was taken and kept on ice to coagulate, centrifuged at 4 °C and snap-frozen in liquid nitrogen directly after. The liver, kidneys, heart and brain were collected and snap-frozen in liquid nitrogen. All samples were stored at −80 °C before analysis.

### Metabolomics in mouse tissues

The mouse body fluid and tissue samples derived from five female *Mmut*-ki/wt and five female *Mmut*-ko/ki mice were collected as described above and prepared as previously published^52^. Sample analysis using LC–MS was performed as previously published^53^. Ions were annotated to metabolites based on exact mass to the KEGG database^54^ considering [M-H+] and 0.01 Da mass accuracy.

### Transcriptomics in mouse brains

Brain tissue samples were collected as described above. Four female mice per genotype groups *Mmut*-ki/wt and *Mmut*-ko/ki were used. RNA was purified using a DNase kit (QIAGEN, 79254) together with QIAmp RNA Blood Mini kit (QIAGEN, 52304). RNA-seq reads were aligned with STAR-aligner^55^. As reference, we used the Ensembl mouse genome build GRCm38. Gene expression values were computed with the function featureCounts from the R package Rsubread^56^.

### CRISPR gene-editing experiments

CRISPR-Cas9 editing was performed in 293T cells (ATCC CRL-3216) as described^57^. Cas9 protein was provided as a plasmid (PX459-V2.0, Addgene, 62988) and guide RNA (MMUT: ATTCCTTTAGTATATCATTT; OGDH: GACTAGTTCGAACTATGTGG; DLST: AACAGGGGAACTGCCCTCTA) as gBLOCKS^58^ (IDT Technologies). The 293T cells were transfected using a Neon transfection system (Thermo Fisher Scientific) containing 100,000 cells, 0.6 µg Cas9 plasmid and 600 ng guide RNA following the manufacturer’s instructions. At 48 h after transfection, cells were collected, diluted to 1 cell per 100 µl and transferred to a 96-well plate at 100 µl per well for clonal selection. Correct clones were confirmed by Sanger sequencing of genomic DNA.

### Western blotting

Protein extraction and Western blotting was performed as described previously^59^. Primary antibodies used were probing for the following proteins: MMUT (Abcam, ab67869, 1:1,000 dilution, mouse host), OGDH (Atlas Antibodies, HPA020347, 1:500 dilution, rabbit host), GLUD (Abcam, ab166618, 1:2,000 dilution, rabbit host) and β-actin (Sigma, A1978, 1:5,000 dilution, mouse host). Secondary antibodies used were anti-rabbit HRP (Santa Cruz, sc-2357, 1:5,000 dilution, mouse host) and anti-mouse HRP (Santa Cruz, sc-516102, 1:5,000 dilution, goat host).

### KGDH enzyme activity assays

An assay of oxoglutarate dehydrogenase enzyme activity was performed in 293T cell clones according to the manufacturer’s instructions (Sigma-Aldrich, MAK189) detected using a VICTOR Nivo system (PerkinElmer).

### Glutamine tracing studies and treatments

The 293T cells or primary fibroblasts were cultured on poly-l-lysine coated coverslips in DMEM with 25 mM glucose and 4 mM l-glutamine (Gibco, 11965092), supplemented with 10% FBS, 1% antibiotic-antimycotic (Gibco). For treatment studies, 1 mM citric acid disodium salt (Sigma, 71635), 6 mM dimethyl 2-oxoglutarate (Sigma, 349631) or 1 mM l-malic acid (Sigma, M7397) was added for 24 h before cell collection. Four hours before cell collection, medium was changed to DMEM with 25 mM glucose without l-glutamine (Gibco, 11960044) supplemented with 10% FBS, 1% antibiotic-antimycotic (Gibco) and 4 mM [U-^13^C] glutamine (Sigma-Aldrich, 605166). At collection, medium was removed, coverslips quickly dipped into sterile double-distilled water at 37 °C and quenched in 80% methanol at −20 °C. Cells were scrapped in methanol and centrifuged at 15,000*g* for 15 min at 4 °C. Supernatants were collected, snap-frozen in liquid nitrogen and stored at −80 °C before LC–MS analysis.

Thawed supernatants were lyophilized overnight and resolubilized in 200 µl loading buffer (water and 0.5% formic acid) in narrow-bottom 96-deep-well plates on a shaker (800 r.p.m., 15 °C, 10 min) for LC–MS injection. Metabolites were separated using an ACQUITY UPLC HSS T3 1.8-µm, 100 × 2.1 mm internal diameter column (Waters) and eluted using the following gradient from solvent A (water, 5 mM ammonium formate and 0.1% formic acid) to solvent B (methanol, 5 mM ammonium formate and 0.1% formic acid) as follows: 2 min at 0% B; 2–3.5 min to 4% B; 3.5–10 min to 45% B; 10–12 min to 70% B; 12–13.5 min to 100% B; with an isocratic plateau at 100% B for 2–15.5 min and from 15.5–16.5 min to 0% B. After each run the column was re-equilibrated for 8 min at 100% A with a constant flow rate of 0.4 ml min^−1^.

Mass spectra were acquired using a heated ESI source of a Q-Exactive high-resolution, accurate mass spectrometer (Thermo Fisher Scientific). Mass spectra were recorded in positive and negative mode with the MS detector in full-scan mode (full MS) in a scan range 50–750 m/z with an AGC target of 1 × 10^6^, an Orbitrap resolution of 70,000 and a maximum injection time of 80 ms. Peaks were integrated with Xcalibur (v.4.0.27.19, Thermo Fisher Scientific) using windows of 0.01 m/z and 20 s for retention time as previously determined using a library of standards. Heated ESI parameters were sheath gas flow rate 35 arbitrary units (AU), auxiliary gas flow rate 35 AU, sweep gas flow rate 2 AU, spray voltage 3.5 kV, capillary temperature 350 °C and aux gas heater temperature 350 °C. Detector settings for full MS were in-source CID 0.0 eV; µscans of 1; resolution of 70,000; AGC target of 1 × 10^6^; max IT of 35 ms and spectrum data type, profile. Integration parameters were ICIS Peak Integration, nearest RT; smoothing points 3; baseline window 40; area noise factor 3; peak noise factor 70; and minimum peak height 3.0. Data preprocessing included missing value imputation and normalization to internal standards [^2^H]_3_-creatine and [^2^H]_4_-citric acid for positive and negative mode, respectively. Experiments were performed in 2–3 clonal replicates, two biological replicates and three technical replicates. Each technical replicate was run in positive and negative mode.

### Affinity-capture mass spectrometry

The 293T cells were grown in DMEM (Gibco) supplemented with 10% fetal bovine serum (Gibco) and antibiotics (GE Healthcare). Transient transfection of each pCDNA3-C-FLAG-LIC construct was performed at least three separate times using Lipofectamine 3000 (Thermo Fisher Scientific) according to manufacturer’s instructions. At 48 h after transfection, cells were crosslinked using 0.5% paraformaldehyde (PFA, Sigma-Aldrich) in PBS (Gibco) for 10 min at RT, the reaction was quenched with 1.25 M glycine/PBS (Sigma-Aldrich) for 10 min at 4 °C, cells were centrifuged for 5 min at 2,000*g* at 4 °C and the pellet resuspended in lysis buffer (1% Nonidet P-40, 0.5% deoxycholine, 150 mM NaCl and 50 mM Tris-HCl, pH 7.5; all Sigma-Aldrich). Pre-cleared cell extracts were immunoprecipitated with anti-FLAG M2 (F3165, Sigma-Aldrich), anti-MMUT (ab67869, Abcam) or anti-MMAB (HPA039017, Sigma-Aldrich) using Dynabeads Protein G (Thermo Fisher Scientific) according to the manufacturer’s instructions.

For affinity capture, all samples were washed with PBS and peptides were released by trypsin (100 ng µl^−1^ in 10 mM HCl) and supernatants were collected, dried and dissolved in 0.1% formic acid. All affinity-captured samples were measured on a Q-Exactive mass spectrometer (Thermo Fisher Scientific) with an MS1 resolution of 70,000, an AGC target of 3 × 10^6^ and a maximum injection time of 100 ms over a scan range of m/z 350–1,500. A data-dependent top-12 method was used for HCD MS/MS with a normalized collision energy of 25 at a resolution of 35,000. Precursor ions were isolated in a 1.2-Th window with an AGC target value of 1 × 10^5^ with a maximum injection time of 120 ms. Dynamic exclusion was set to 40 s.

Samples were analyzed using Mascot (Matrix Science, v.2.6.2) with the SwissProt database (downloaded 4 February 2019) assuming trypsin with at maximum two miscleavages. Mascot was searched with a fragment ion mass tolerance of 0.030 Da and a parent ion tolerance of 10.0 ppm. Oxidation of methionine was specified as a variable modification. Scaffold (v.Scaffold_5.1.2, Proteome Software) was used to validate MS/MS-based peptide and protein identifications. Peptide identifications were accepted if they could be established at greater than 95.0% probability by the Scaffold Local FDR algorithm. Protein identifications were accepted if they could be established at greater than 99.0% probability and contained at least two identified peptides.

For immunoblotting, the procedure was the same as described above with the exception that samples were detected using anti-FLAG (1:2,000 dilution; Sigma-Aldrich), anti-MMUT (1:500 dilution, Abcam), anti-MMAB (1:1,000 dilution, Sigma-Aldrich) or anti-DLST (1:1000 dilution, D22B1, Cell Signaling Technology) primary antibodies. Indicated proteins were detected by HRP-labeled anti-mouse (ab131368, Abcam) or anti-rabbit (ab131366, Abcam) secondary antibodies at a dilution of 1:5,000.

### Genetic variant investigation approach

Short-variant calling was carried out with GATK and DeepVariant algorithms and annotated with annovar^60^. Copy-number variations (CNVs) were called with CNVnator^61^ with a bin size of 100 and standard parameters and annotated with AnnotSV^62^. Variation in the *MMUT* gene was investigated first. When no genetic cause for the phenotype was identified with this approach (two inactivating/damaging events in *MMUT*), other genes known to be involved in MMA (based on literature reports) were investigated as a virtual gene panel. When no genetic cause was found in the two previous steps, genes highlighted by mutational burden (genes harboring pathogenic variants across the cohort in an autosomal recessive pattern in two or more individuals) were investigated. Finally, all samples and controls were used to run OUTRIDER^21^ and genes highlighted as expression outliers associated with phenotypes overlapping MMA were analyzed to either confirm the identified damaging variants, or to further explore damaging variation in them.

Variants were prioritized with the following approach: First, any coding variant (excluding synonymous variants) with a GnomAD frequency across all represented populations <0.01, in homozygosity or compound heterozygosity with another relevant variant and supported by at least two forward and two reverse reads and at least eight reads coverage, were evaluated. Second, all variants categorized by the automatic application of the ACMG criteria^63^ by InterVar^64^ or classified in ClinVar^65^ as ‘pathogenic’ or ‘likely pathogenic’, in homozygosity or compound heterozygosity with another relevant variant, were considered and evaluated. Third, variants with a dbscSNV_ADA or dbscSNV_RF scores > 0.6 in the annovar annotation using the database prepared and described previously^66^ were evaluated.

For CNVs, individuals with a single heterozygous variant or no variation in *MMUT* and the other genes of interest were investigated for the presence of relevant CNVs that could explain their phenotype^60^.

### Data analysis

For differential expression analysis, we quantile normalized the response variable (PI activity measures) to have it follow a standard normal distribution and ensure that the normality assumption holds. Proteomics and RNA-seq expression matrices were iteratively column- and row-wise standardized to ensure mean zero and unit variance both row- and column-wise^67,68^. We then ran a mixed model with the gene expression vector being used as a fixed effect and a random effect with the same covariance structure as the expression data after column and row normalization as described before^25^. For the global data layer inspection we used the MOFA v.1.3.1 (ref. ^23^), MASS v.7.3-54 (ref. ^69^) and fgsea v.1.18.0 (ref. ^70^) packages. MOFA was run on log-transformed data. Gene enrichment analysis was performed using gene sets downloaded from http://www.gsea-msigdb.org/gsea/msigdb/index.jsp ‘MSigDB Collections’ on 28 December 2020. Circos, including chord plots, were created using the circlize package v.0.4.13 (ref. ^71^). The UniProt portal was accessed on 24 February 2021 to scrape protein localization data. Data analysis was performed using R v.4.1.0.

### Ethical compliance

Collection and use of primary fibroblast cells and informed consent for phenotypic data were performed as approved by the Ethics Committee of the Canton of Zurich (KEK-2014–0211, amendment PB_2020-00053). All animal experiments were approved by the Cantonal Veterinary Office Zurich (license no. 202/2014).

### Reporting summary

Further information on research design is available in the Nature Portfolio Reporting Summary linked to this article.

## Supplementary information

Supplementary Information Supplementary Figs. 1–6, Supplementary Tables 1 and 2 and Supplementary Document 1.

Reporting Summary

## Data Availability

Access to the raw genomic and transcriptomic data is restricted due to ethical concerns. Data can be made available upon reasonable request to D.S.F. within 3 months following an established data transfer, use agreement and ethical approval. The MS proteomics data (.raw files) have been deposited to the ProteomeXchange Consortium (http://proteomecentral.proteomexchange.org) via the MassIVE partner repository (https://massive.ucsd.edu) with dataset identifiers MSV000088791 and PXD038225. Metabolomics MS raw data for human fibroblast measurements have been uploaded to the MassIVE data repository (https://massive.ucsd.edu) with dataset identifier MSV000089082. IP-MS raw files have been deposited to the ProteomeXchange Consortium via the MassIVE partner repository (https://massive.ucsd.edu) with dataset identifier MSV000088791. Source data are provided with this paper.

## Source data

Source Data Fig. 1 Transcriptomics and proteomics source data.
Source Data Fig. 2 Raw phenotype data.
Source Data Fig. 3 RNA-seq mouse statistical source data.
Source Data Fig. 5 Metabolomics source data.
Source Data Fig. 6 Unprocessed pull-down data.
Source Data Fig. 6 Unprocessed Western blots.
Source Data Extended Data Fig. 9 Unprocessed Western blots.
Source Data Extended Data Fig. 10 Unprocessed Western blots.

## Code availability

Computer code for data analyses is hosted on a repository on the GitHub platform and accessible at https://github.com/pforny/MMAomics.
