# Supplementary material for: Integrated multi-omics reveals anaplerotic rewiring in methylmalonyl-CoA mutase deficiency
Source: Nat Metab. 2023 Jan 26;5(1):80–95. doi: 10.1038/s42255-022-00720-8 (PMC9886552; doi:10.1038/s42255-022-00720-8)
Supplement: Supplementary file 1 — Supplementary Figs. 1–6, Supplementary Tables 1 and 2 and Supplementary Document 1. [file 42255_2022_720_MOESM1_ESM.pdf]

nature metabolism

Article https://doi.org/10.1038/s42255-022-00720-8

Integrated multi-omics reveals anaplerotic rewiring in methylmalonyl-CoA mutase deficiency

In the format provided by the authors and unedited

Supplementary Figure 1. Phenotype data analysis performed on the complete set of variables.

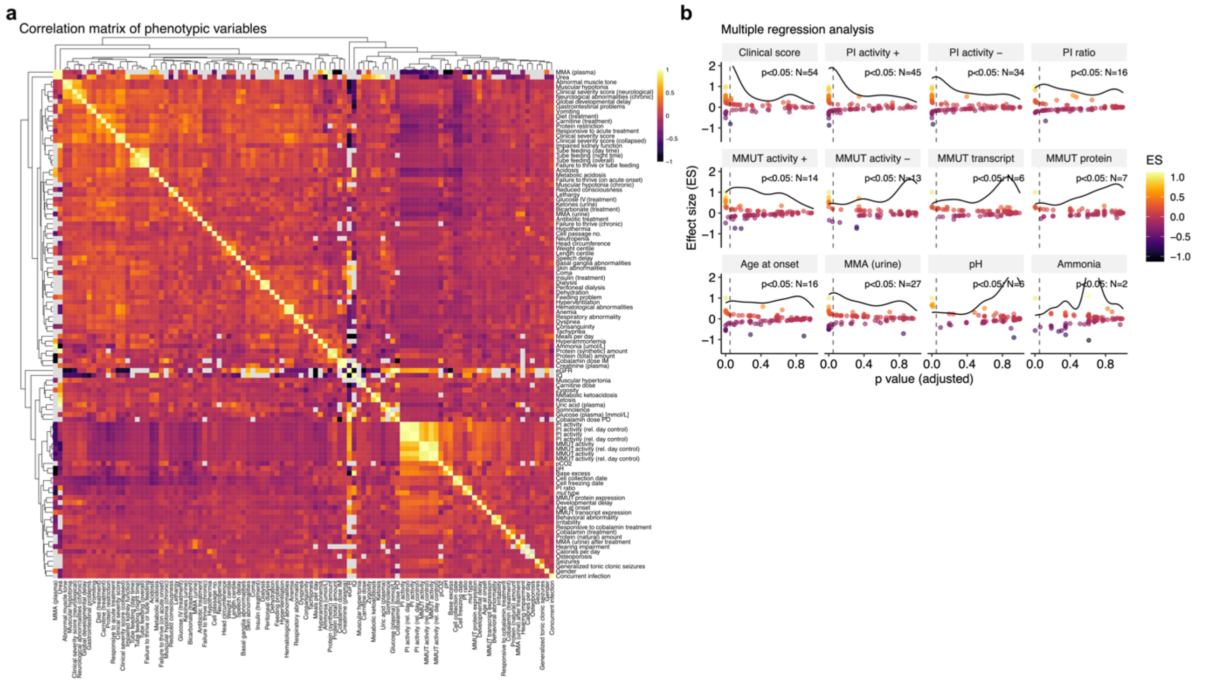

This figure represents the same analysis as performed in Figure 2 of the main manuscript, which shows the phenomics analysis excluding nonspecific clinical symptoms. Here, the complete set of phenotypic variables is used as available from **Source Data Table 1**. **a**, Correlation matrix of all continuous numeric and discrete phenotype variables. **b**, Panel of selected phenotypic traits and their overall strength of representing the entirety of the phenomics dataset (here termed disease severity) as assessed by linear modeling after log transformation. Each point represents the result of linear regression against one other phenotypic variable with the effect size on the y-axis and the resulting Benjamini-Hochberg adjusted p-value on the x-axis. The horizontal curved line indicates the density of data points as distributed along the x-axis. The vertical dashed line indicates the threshold of significance (p-value <0.05).

Supplementary Figure 2. Global computational approaches to transcriptomics and proteotyping datasets were unable to stratify samples into disease and non-disease groups.

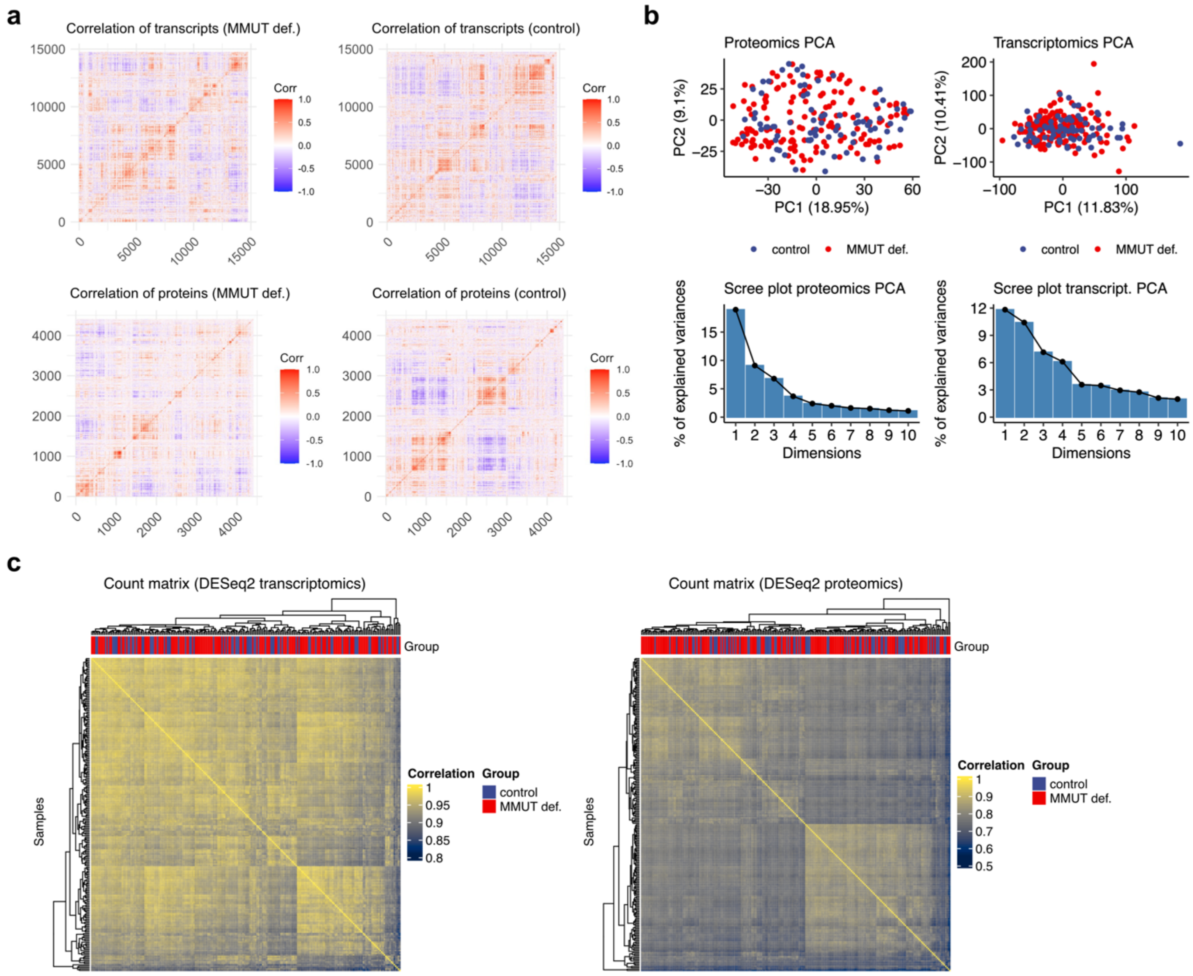

a, Pearson correlation matrices of all transcripts and proteins grouped by MMUT-deficient and control groups. **b**, Principal component analysis of transcriptomics and proteomics datasets with prior gene- and sample-wise scaling. **c**, Quality control heatmap of the differential expression analysis using the DESeq2 package.

Supplementary Figure 3. Transcriptomics analysis of mouse brain revealed sample clustering according to genotype.

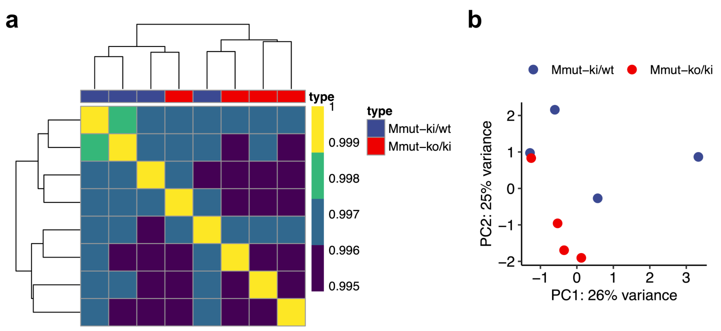

a, Quality control heatmap and, **b**, PCA plot based on differential expression analysis performed by applying the DESeq2 R package.

Supplementary Figure 4. Significantly dysregulated proteins were enriched for mitochondrial localization.

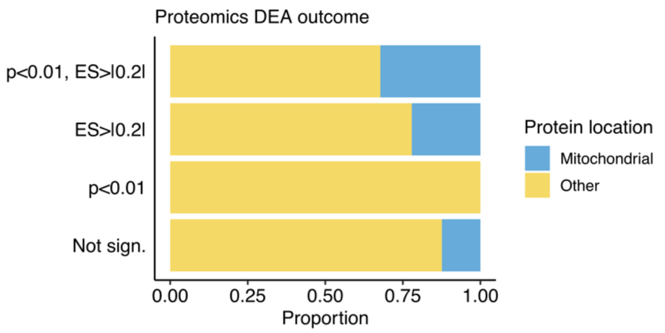

Proportions of proteins localized to the mitochondria (according to MitoCarta 3.0) split according to the result of the differential expression analysis (Fig. 4b); ES, effect size.

Supplementary Figure 5. Validation of CRISPR knock-out 293T cell lines.

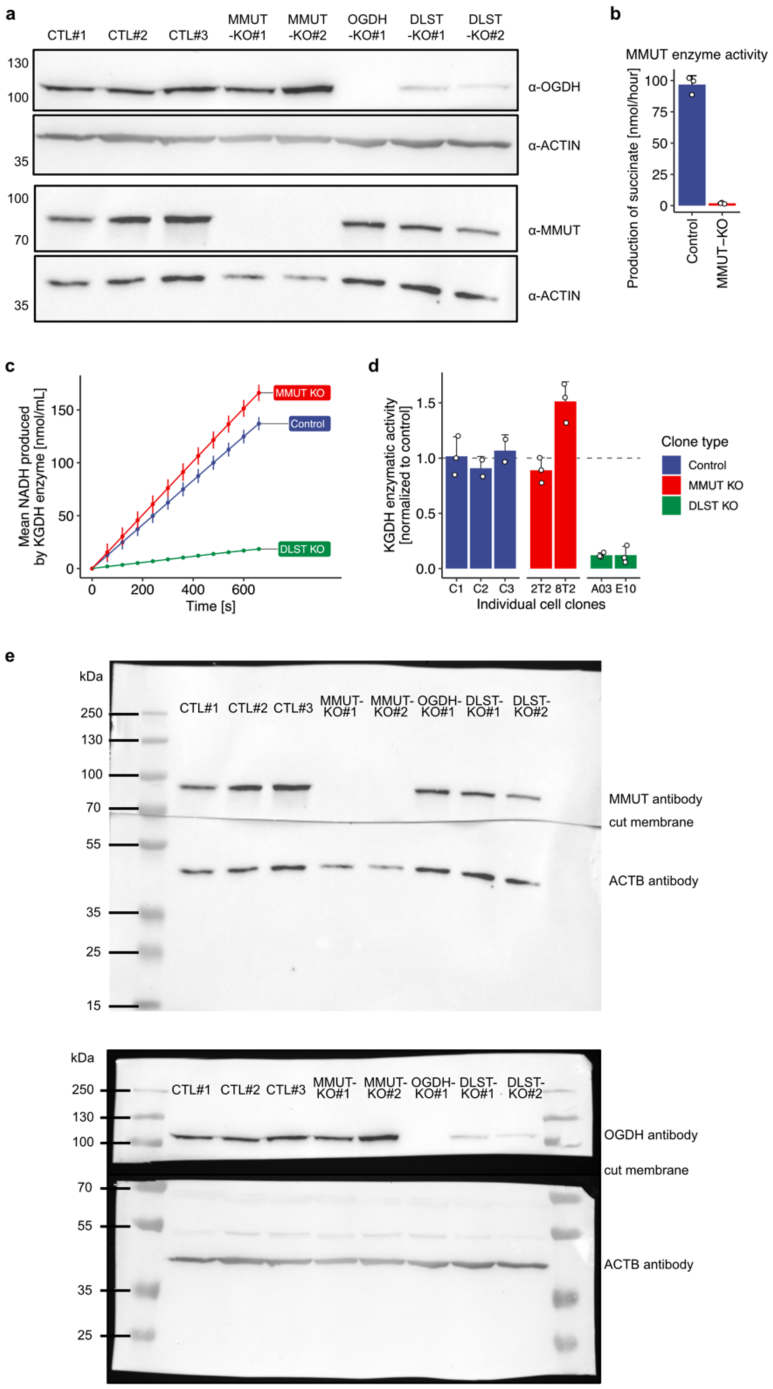

a, Western blots probing for OGDH and MMUT in CRISPR knock-out cell lines. Cell line numbers indicate biological replicates; *OGDH*-KO cell line was not used in this study. **b**, MMUT enzymatic activity assessed by succinate production; data points indicate means of n=3 independent experiments; error bars indicate SD, centered around the mean. **c**, Alpha-ketoglutarate dehydrogenase (KGDH) enzyme activity assessed by spectrophotometric measurement of produced NADH over time; colors indicate genetic background of cells, consisting of n=3 (control) and n=2 (MMUT-KO and DLST-KO) biological replicates, respectively; data from 3 independent experiments per biological replicate; error bars indicate +/- SD, centered around the mean calculated per time point. **d**, Enzyme activities in

individual clones normalized to the first control (wildtype) cell line (C1); each bar represents a biological replicate, dots indicate n=3 independent experiments; error bars indicate SD, centered around the mean. **e**, Raw Western blot images shown in a.

Supplementary Figure 6. Metabolomics and glutamine labelling in 293T cells.

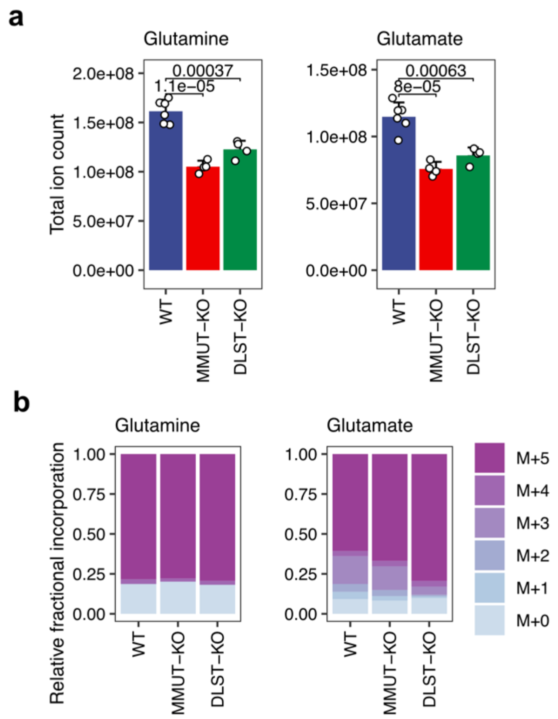

a, Pool sizes of metabolites in control and CRISPR/Cas9 KO 293T cells; n=3 biologically independent samples (WT), n=2 (MMUT-KO), n=2 (DLST-KO) over 2 independent experiments were measured; error bars represent SD, centered around the mean; p-values calculated by t-test, two-sided. **b**, Fractional labelling of glutamine and glutamate upon tracing with [U-^{13}C]glutamine.

Supplementary Table 1. Overview of pathogenic variants.

Variants detected on WGS (novel variants in bold); nt, nucleotide; aa, amino acid.

Cell line number	Forny et al. number (PMID: 27167370)	Gene	Variant 1 (nt)	Variant 1 (aa)	Variant 2 (nt)	Variant 2 (aa)	Comment
MMA001	2	MMUT	c.654A>C	p.Gln218His	c.1106G>A	p.Arg369His	
MMA002	3	MMUT	c.1106G>A	p.Arg369His	c.1106G>A	p.Arg369His	
MMA003	6	MMUT	c.409C>T	**p.Ala137Val**	c.655A>T	p.Asn219Tyr	
MMA004	7	MMUT	c.607G>A	p.Gly203Arg	c.1106G>A	p.Arg369His	
MMA005	8	MMUT	c.982C>T	p.Leu328Phe	c.982C>T	p.Leu328Phe	
MMA006	9	MMUT	c.1106G>A	p.Arg369His	c.691T>A	p.Tyr231Asn	
MMA007	10	MMUT	c.1106G>A	p.Arg369His	c.1106G>A	p.Arg369His	
MMA008	11	MMUT	c.1031C>T	**p.Ser344Phe**	c.312delC	p.Trp105Glyfs*75	
MMA009	12	MMUT	c.607G>A	p.Gly203Arg	c.607G>A	p.Gly203Arg	
MMA010	13	MMUT	c.299A>G	p.Tyr100Cys	c.299A>G	p.Tyr100Cys	
MMA011	14	MMUT	c.1361G>A	**p.Gly454Glu**	c.427C>T	**p.His143Tyr**	
MMA012	15.1	MMUT	c.1106G>A	p.Arg369His	c.1097A>G	**p.Asn366Ser**	
MMA013	16	MMUT	c.2081G>T	**p.Arg694Leu**	c.1207C>T	p.Arg403*	
MMA014	20	MMUT	c.862T>C	**p.Ser288Pro**	c.862T>C	**p.Ser288Pro**	
MMA015	21	MMUT	c.2080C>T	p.Arg694Trp	c.2080C>T	p.Arg694Trp	
MMA016	22	MMUT	c.2099T>A	p.Met700Lys	c.623_624delTA	**p.Val208Alafs*2**	
MMA017	24	MMUT	c.655A>T	p.Asn219Tyr	c.655A>T	p.Asn219Tyr	
MMA018	25	MMUT	c.655A>T	p.Asn219Tyr	c.1420C>T	p.Arg474*	
MMA019	26	MMUT	c.443C>T	**p.Ser148Leu**	c.1677-1G>C	Splice site	
MMA020	32	MMUT	c.655A>T	p.Asn219Tyr	c.1560+1G>T	Splice site	
MMA021	33	MMUT	c.655A>T	p.Asn219Tyr	c.1889G>A	p.Gly630Glu	
MMA022	34	MMUT	c.1106G>A	p.Arg369His	c.2009delG	**p.Gly670Alafs*2**	
MMA023	36	MMUT	c.572C>A	p.Ala191Glu	c.572C>A	p.Ala191Glu	
MMA024	38	MMUT	c.2159-2160delAT	**p.Asn720Serfs*17**	c.2159-2160delAT	p.Asn720Serfs*736	
MMA025	38.1	MMUT	c.655A>T	p.Asn219Tyr	c.655A>T	p.Asn219Tyr	
MMA026	41	MMUT	c.566A>T	p.Asn189Ile	c.1658delT	p.Val553Glyfs*17	
MMA027	44	MMUT	c.88C>T	p.Gln30*	c.88C>T	p.Gln30*	
MMA028	45	MMUT	c.654A>C	p.Gln218His	c.654A>C	p.Gln218His	
MMA029	46	MMUT	c.1962_1963delTC	p.Arg655*	c.1962_1963delTC	p.Arg655*	
MMA030	47	MMUT	c.689C>G	p.Thr230Arg	c.689C>G	p.Thr230Arg	
MMA031	49	MMUT	c.1084-10A>G	p.Gln361_Asp362insIlePhe*	c.1084-10A>G	p.Gln361_Asp362insIlePhe*	
MMA032	50	MMUT	c.786T>G	**p.Ser262Arg**	c.1889G>A	p.Gly630Glu	
MMA033	51	MMUT	c.-39-1G>A	splice site	c.-39-1G>A	Splice site	
MMA034	52	MMUT	c.914T>C	p.Leu305Ser	c.914T>C	p.Leu305Ser	
MMA035	54	MMUT	c.378C>A	p.Asn126Lys	c.974G>A	p.Gly325Asp	
MMA036	55	MMUT	c.2179C>T	p.Arg727*	c.2179C>T	p.Arg727*	
MMA037	57	MMUT	c.851G>A	p.Gly284Glu	c.982C>T	p.Leu328Phe	
MMA038	58	MMUT	c.572C>A	p.Ala191Glu	c.1541delA	**p.Gln514Argfs*24**	
MMA039	59	MMUT	c.1874A>T	**p.Asp625Val**	c.1874A>T	**p.Asp625Val**	
MMA040	60	MMUT	c.421delG	p.Ala141Argfs*39	c.421delG	p.Ala141Argfs*39	
MMA041	61	MMUT	c.1843C>A	p.Pro615Thr	c.2179C>T	p.Arg727*	
MMA042	62	MMUT	c.692dupA	**p.Tyr231***	c.692dupA	**p.Tyr231***	
MMA043	63	MMUT	c.982C>T	p.Leu328Phe	c.982C>T	p.Leu328Phe	
MMA044	67	MMUT	c.753+2T>A	Splice site	c.2206C>T	p.Leu736Phe	
MMA045	74	MMUT	c.1276G>A	p.Gly426Arg	c.1655C>T	p.Ala552Val	
MMA046	101	MMUT	c.654A>C	p.Gln218His	c.1106G>A	p.Arg369His	
MMA047	121	MMUT	c.1160C>T	**p.Thr387Ile**	c.1160C>T	**p.Thr387Ile**	
MMA048	125	MMUT	c.655A>T	p.Asn219Tyr	c.828G>C	p.Glu276Asp	
MMA049	137	MMUT	c.977G>A	p.Arg326Lys	c.2194_2197delinsTGGAA	p.Ala762Trpfs*6	
MMA050	138	MMUT	c.683G>A	**p.Arg228Gln**	c.2200C>T	p.Gln734*	
MMA051		MMUT	c.1022dupA	p.Asn341fs*	c.2150G>T	p.Gly717Val	
MMA052		MMUT	c.2080C>T	p.Arg694Trp	c.2080C>T	p.Arg694Trp	

MMA053		MMUT	c.277C>T	p.Arg93Cys	c.1207C>T	p.Arg403*	
MMA054		MMUT	c.91C>T	p.Arg31*	c.323G>A	p.Arg108His	
MMA055		MMUT	c.662T>A	**p.Ile221Lys**	c.1790_1791insCT	**p.Thr598***	
MMA056		MMUT	c.1885A>G	p.Arg629Gly	c.1885A>G	p.Arg629Gly	
MMA057		MMUT	**c.1560+3A>G**	Splice site	**c.1560+3A>G**	Splice site	
MMA058		MMUT	c.850G>A	p.Gly284Arg	c.1073T>C	**p.Leu358Pro**	
MMA059		MMUT	c.544dupA	**p.Met182Asnfs*29**	c.544dupA	**p.Met182Asnfs*29**	
MMA060		MMUT	**c.1560+3A>G**	Splice site	**c.1560+3A>G**	Splice site	
MMA061		MMUT	c.2179C>T	p.Arg727*	c.2179C>T	p.Arg727*	
MMA062		MMUT	c.655A>T	p.Asn219Tyr	c.1055dupA	**p.Thr353Aspfs***	
MMA063		MMUT	c.1846C>T	p.Arg616Cys	c.1588_1595delGCTGAACG	**p.Ala530Leufs*11**	
MMA064		MMUT	c.2080C>T	p.Arg694Trp	c.360dupT	p.Lys121*	
MMA065		MMUT	c.-39-1G>A	splice site	c.-39-1G>A	Splice site	
MMA066		MMUT	c.1531C>T	p.Arg511*	c.1531C>T	p.Arg511*	
MMA067		MMUT	c.1843C>A	p.Pro615Thr	c.1843C>A	p.Pro615Thr	
MMA068		MMUT	c.329A>G	p.Tyr110Cys	c.329A>G	p.Tyr110Cys	
MMA069		MMUT	c.160A>T	p.Lys54*	c.160A>T	p.Lys54*	
MMA070		MMUT	c.521T>C	p.Phe174Ser	c.521T>C	p.Phe174Ser	
MMA071		MMUT	c.1181T>A	**p.Leu394***	c.91C>T	p.Arg31*	
MMA072		MMUT	c.1207C>T	p.Arg403*	c.572C>A	p.Ala191Glu	
MMA073		MMUT	c.1808G>A	p.Arg603Lys	c.1808G>A	p.Arg603Lys	
MMA074		MMUT	c.2115dupA	**p.Pro706Thrfs*6**	c.2115dupA	**p.Pro706Thrfs*6**	
MMA075		MMUT	c.-39-1G>A	Splice site	c.91C>T	p.Arg31*	
MMA076		MMUT	c.91C>T	p.Arg31*	c.91C>T	p.Arg31*	
MMA077		MMUT	c.1106G>A	p.Arg369His	het deletion of exon 4		
MMA078		MMUT	c.597T>C	p.Phe174Ser	c.597T>C	p.Phe174Ser	
MMA079		MMUT	c.1889G>A	p.Gly630Glu	c.1889G>A	p.Gly630Glu	
MMA080		MMUT	c.572C>A	p.Ala191Glu	c.572C>A	p.Ala191Glu	
MMA081		MMUT	c.2179C>T	p.Arg727*	c.1106G>A	p.Arg369His	
MMA082		MMUT	c.1531C>T	p.Arg511*	c.1531C>T	p.Arg511*	
MMA083		MMUT	c.330T>G	p.Tyr110*	c.164delA	**p.Asn55Thrfs*5**	
MMA084		MMUT	c.1962_1963delTC	p.Arg655*	c.1962_1963delTC	p.Arg655*	
MMA085		MMUT	c.1690G>T	**p.Glu564***	c.1690G>T	**p.Glu564***	
MMA086		MMUT	c.655A>T	p.Asn219Tyr	c.655A>T	p.Asn219Tyr	
MMA087		MMUT	c.1844C>T	p.Pro615Leu	c.1844C>T	p.Pro615Leu	
MMA088							
MMA089		MMUT	c.572C>A	p.Ala191Glu	c.2194_2197delinsTGGAA	p.Ala762Trpfs*6	
MMA090		MMUT	c.1843C>A	p.Pro615Thr	c.1843C>A	p.Pro615Thr	
MMA091		MMUT	c.1240G>T	p.Glu414*	c.1240G>T	p.Glu414*	
MMA092		MMUT	c.1207C>T	p.Arg403*	c.1207C>T	p.Arg403*	
MMA093		MMUT	c.1311_1312insA	**p.Val438Serfs*3**	c.1311_1312insA	**p.Val438Serfs*3**	
MMA094		MMUT	c.655A>T	p.Asn219Tyr	c.1782_1786delTAAAG	**p.Ser594Argfs*11**	
MMA095		MMUT	c.655A>T	p.Asn219Tyr	c.322C>T	p.Arg108Cys	
MMA096		MMUT	c.394C>T	p.Gln132*	c.394C>T	p.Gln132*	
MMA097		MMUT	c.1843C>A	p.Pro615Thr	c.1843C>A	p.Pro615Thr	
MMA098		MMUT	c.1880A>G	p.His627Arg	c.654A>C	p.Gln218His	
MMA099		MMUT	c.647C>T	**p.Thr216Ile**	c.C647T	**p.Thr216Ile**	
MMA100		MMUT	c.420C>T	p.Arg474*	c.753+2T>A	Splice site	
MMA101		MMUT	c.1280G>A	p.Gly427Asp	c.323G>A	p.Arg108His	
MMA102		MMUT	c.1280G>A	p.Gly427Asp	c.729_730insTT	p.Asp244Leufs*	
MMA103		MMUT	c.654A>C	p.Gln218His	c.654A>C	p.Gln218His	
MMA104		MMUT	c.454C>T	p.Arg152*	c.454C>T	p.Arg152*	
MMA105		MMUT	c.884G>T	**p.Gly295Val**	c.884G>T	**p.Gly295Val**	
MMA106		MMUT	**c.1560+3A>G**	Splice site	**c.1560+3A>G**	Splice site	
MMA107		MMUT	c.1808G>A	p.Arg603Lys	c.1808G>A	p.Arg603Lys	
MMA108		MMUT	c.454C>T	p.Arg152*	c.454C>T	p.Arg152*	

MMA109		MMUT	c.1670G>C	**p.Arg557Pro**	c.1207C>T	p.Arg403*	
MMA110		MMUT	c.982C>T	p.Leu328Phe	c.360dupT	p.Lys121*	
MMA111		MMUT	c.146dupA	**p.Gln50Alafs***	c.146dupA	**p.Gln50Alafs***	
MMA112		MMUT	c.1399C>T	p.Arg467*	c.1399C>T	p.Arg467*	
MMA113		MMUT	c.1880A>G	p.His627Arg	c.655A>T	p.Asn219Tyr	
MMA114		MMUT	c.394C>T	p.Gln132*	c.323G>T	**p.Arg108Leu**	
MMA115		MMUT	c.160A>T	p.Lys54*	c.160A>T	p.Lys54*	
MMA116		MMUT	c.2080C>T	p.Arg694Trp	**c.754-5T>G**	Splice site	
MMA117		MMUT	c.1843C>A	p.Pro615Thr	c.1843C>A	p.Pro615Thr	
MMA118		MMUT	c.129G>A	p.Trp43*	c.129G>A	p.Trp43*	
MMA119		MMUT	c.88C>T	p.Gln30*	c.88C>T	p.Gln30*	
MMA120		MMUT	c.1399C>T	p.Arg467*	c.323G>A	p.Arg108His	
MMA121		MMUT	c.278G>A	p.Arg93His	c.278G>A	p.Arg93His	
MMA122		MMUT	c.129G>A	p.Trp43*	het deletion of exon 4		
MMA123		MMUT	c.655A>T	p.Asn219Tyr	c.655A>T	p.Asn219Tyr	
MMA124		MMUT	c.1846C>T	p.Arg616Cys	c.1846C>T	p.Arg616Cys	
MMA125		MMUT	c.1106G>A	p.Arg369His	c.1106G>A	p.Arg369His	
MMA126							no coverage of the MMUT gene (homozygous deletion of 170 kB), clear cut to the surrounding regions which are well covered
MMA127		MMUT	c.1918G>T	**p.Asp640Tyr**	c.1912T>A	**p.Phe638Ile**	
MMA128		MMUT	c.C1843A	p.Pro615Thr	c.C1420T	p.Arg474*	
MMA129		MMUT	c.2T>C	p.Met1Thr	c.2T>C	p.Met1Thr	
MMA130		MMUT	c.682C>T	p.Arg228*	c.88C>T	p.Gln30*	
MMA131		MMUT	c.1677-1G>C	Splice site	c.1677-1G>C	Splice site	
MMA132		MMUT	c.422C>A	p.Ala141Glu	c.323G>A	p.Arg108His	
MMA133		MMUT	c.421delG	p.Ala141Argfs*39	c.421delG	p.Ala141Argfs*39	
MMA134		MMUT	c.360dupT	p.Lys121*	c.360dupT	p.Lys121*	
MMA135		MMUT	c.323G>A	p.Arg108His	c.1758delA	**p.Tyr587Ilefs*11**	
MMA136		MMUT	c.1843C>A	p.Pro615Thr	c.1843C>A	p.Pro615Thr	
MMA137		MMUT	c.643G>A	p.Gly215Ser	c.454C>T	p.Arg152*	
MMA138		MMUT	c.1106G>A	p.Arg369His	c.1560+1G>T	Splice site	
MMA139							
MMA140		MMUT	c.278G>A	p.Arg93His	c.278G>A	p.Arg93His	
MMA141		MMUT	c.1207C>T	p.Arg403*	c.1207C>T	p.Arg403*	
MMA142		MMUT	c.1843C>A	p.Pro615Thr	c.1843C>A	p.Pro615Thr	
MMA143		MMUT	c.1038_1040delTCT	p.Leu347del	c.1038_1040delTCT	p.Leu347del	
MMA144		MMUT	c.397G>A	p.Gly133Arg	c.397G>A	p.Gly133Arg	
MMA145		MMUT	c.572C>A	p.Ala191Glu	c.572C>A	p.Ala191Glu	
MMA146		MMUT	c.1105C>T	p.Arg369Cys	c.91C>T	p.Arg31*	
MMA147		MMUT	c.2080C>T	p.Arg694Trp	c.1677-1G>C	Splice site	
MMA148		MMUT	**c.1444+2T>G**	Splice site	c.839dupC	**p.Leu281Phefs*9**	
MMA149		MMUT	c.1240G>T	p.Glu414*	c.1240G>T	p.Glu414*	
MMA150		MMUT	c.1962_1963delTC	p.Arg655*	c.1962_1963delTC	p.Arg655*	
MMA151		ACSF3	c.1066G>A	p.Gly356Ser	c.1066G>A	p.Gly356Ser	
MMA152		MMAB	expression outlier				cblB by complementation
MMA153							
MMA154		ACSF3	c.401T>C	p.Leu134Pro	c.401T>C	p.Leu134Pro	
MMA155		ACSF3	c.1412G>A	p.Arg471Gln	c.1412G>A	p.Arg471Gln	
MMA156		ACSF3	c.1A>G	p.Met1Val	c.1A>G	p.Met1Val	
MMA157							
MMA158		ACSF3	c.1672C>T	p.Arg558Trp			
MMA159							
MMA160		ACSF3	c.1672C>T	p.Arg558Trp	c.1075G>A	p.Glu359Lys	
MMA161							
MMA162							
MMA163							
MMA164		SUCLA2	c.534+1G>A	Splice site	c.534+1G>A	Splice site	
MMA165		ACSF3	c.1470G>C	p.Glu490Asp	c.1470G>C	p.Glu490Asp	

MMA166							
MMA167							
MMA168							
MMA169							
MMA170		ACSF3	c.1543C>T	p.Arg515Trp	c.1672C>T	p.Arg558Trp	
MMA171							
MMA172							
MMA173		ACSF3	c.1614-2A>G	Splice site	c.1613+3A>C	splice site	
MMA174							
MMA175		ACSF3	c.1412G>A	p.Arg471Gln	c.1412G>A	p.Arg471Gln	
MMA176		TCN2	c.172delC	p.Leu58Tyrfs *28	c.172delC	p.Leu58Tyrfs *28	
MMA177							
MMA178							
MMA179		ACSF3	c.1470G>C	p.Glu490Asp	c.1470G>C	p.Glu490Asp	
MMA180							
MMA181							
MMA182		ACSF3	c.1672C>T	p.Arg558Trp	c.1672C>T	p.Arg558Trp	
MMA183		SUCLA2	c.664-1G>A	Splice site	c.664-1G>A	Splice site	
MMA184							
MMA185							
MMA186		ACSF3	expression outlier				
MMA187		ACSF3	expression outlier				
MMA188							
MMA189		ACSF3	c.1672C>T	p.Arg558Trp	c.1075G>A	p.Glu359Lys	
MMA190							
MMA191							
MMA192		SUCLA2	c.1106dupA	p.Val370Glyf s*16			
MMA193		ACSF3	c.1470G>C	p.Glu490Asp	c.1470G>C	p.Glu490Asp	
MMA194							
MMA195		TCN2	c.328dupC	p.Leu110Prof s*8	c.328dupC	p.Leu110Prof s*8	
MMA196		ACSF3	c.1672C>T	p.Arg558Trp	c.1672C>T	p.Arg558Trp	
MMA197		ACSF3	c.1672C>T	p.Arg558Trp	c.1672C>T	p.Arg558Trp	
MMA198		TCN2	c.497_498del TC	p.Leu166Prof s*7	c.1137_1138i nsA	p.Tyr380Ilefs *32	
MMA199							
MMA200							
MMA201							
MMA202		ACSF3	c.1672C>T	p.Arg558Trp	c.1672C>T	p.Arg558Trp	
MMA203		ACSF3	c.1672C>T	p.Arg558Trp	c.1672C>T	p.Arg558Trp	
MMA204							
MMA205							
MMA206		MMAA	expression outlier				cblA by complementation
MMA207							
MMA208							
MMA209		MMAA	expression outlier				MMAA cDNA transcript not amplifiable
MMA210							cblB by complementation
MMA211							
MMA212							
MMA213							
MMA214							
MMA215							
MMA216							
MMA217							
MMA218							
MMA219							
MMA220							
MMA221							
MMA222							
MMA223							
MMA224							
MMA225							
MMA226							
MMA227							

MMA228							
MMA229							
MMA230							

Supplementary Table 2. List of significantly enriched proteins in pull-down by affinity capture mass spectrometry using MMUT, MMAB, or MCEE as baits.

Accession Number	Molecular Weight	ANOVA Test (p-value)	Quantitative Profile
**MMUT-pulldown**			
AATM_HUMAN	48 kDa	0.0001	EV_flag low, MUT_flag high, VLCAD_flag low
CH10_BOVIN (+1)	11 kDa	0.00022	EV_flag low, MUT_flag high, VLCAD_flag low
ODPB_HUMAN	39 kDa	0.0012	EV_flag low, MUT_flag high, VLCAD_flag low
MUTA_HUMAN [3]	83 kDa	0.0014	EV_flag low, MUT_flag high, VLCAD_flag low
GRPE1_HUMAN (+1)	24 kDa	0.0039	EV_flag low, MUT_flag high, VLCAD_flag low
P5CR1_HUMAN	33 kDa	0.0039	EV_flag low, MUT_flag high, VLCAD_flag low
ATP5H_HUMAN	18 kDa	0.0039	EV_flag low, MUT_flag high, VLCAD_flag low
EFTU_HUMAN	50 kDa	0.0042	EV_flag low, MUT_flag high, VLCAD_flag low
P5CS_HUMAN	87 kDa	0.013	EV_flag low, MUT_flag high, VLCAD_flag low
MDHM_HUMAN	36 kDa	0.015	EV_flag low, MUT_flag high, VLCAD_flag low
SSBP_HUMAN	17 kDa	0.019	EV_flag low, MUT_flag high, VLCAD_flag low
GLYM_HUMAN [2]	56 kDa	0.021	EV_flag low, MUT_flag high, VLCAD_flag low
ATPO_HUMAN	23 kDa	0.022	EV_flag low, MUT_flag high, VLCAD_flag low
RT23_HUMAN	22 kDa	0.027	EV_flag low, MUT_flag high, VLCAD_flag low
RT27_HUMAN	48 kDa	0.027	EV_flag low, MUT_flag high, VLCAD_flag low
STML2_HUMAN	39 kDa	0.031	EV_flag low, MUT_flag high, VLCAD_flag low
ATPG_HUMAN	33 kDa	0.031	EV_flag low, MUT_flag high, VLCAD_flag low
ODO2_HUMAN	49 kDa	0.034	EV_flag low, MUT_flag high, VLCAD_flag low
IDH3A_HUMAN	40 kDa	0.034	EV_flag low, MUT_flag high, VLCAD_flag low
ETFB_HUMAN	28 kDa	0.046	EV_flag low, MUT_flag high, VLCAD_flag low
C1QBP_HUMAN	31 kDa	0.046	EV_flag low, MUT_flag high, VLCAD_flag low
DHE3_HUMAN [2]	61 kDa	0.048	EV_flag low, MUT_flag high, VLCAD_flag low
**MMAB-pulldown**			
MMAB_HUMAN	27 kDa	0.0001	EV_flag low, MMAB_flag high, VLCAD_flag low
CH10_BOVIN (+1)	11 kDa	0.0001	EV_flag low, MMAB_flag high, VLCAD_flag low
DHE3_HUMAN [2]	61 kDa	0.00029	EV_flag low, MMAB_flag high, VLCAD_flag low
ATPO_HUMAN	23 kDa	0.00069	EV_flag low, MMAB_flag high, VLCAD_flag low
P5CR1_HUMAN	33 kDa	0.0012	EV_flag low, MMAB_flag high, VLCAD_flag low
ETFB_HUMAN	28 kDa	0.0018	EV_flag low, MMAB_flag high, VLCAD_flag low
SYDM_HUMAN	74 kDa	0.0024	EV_flag low, MMAB_flag high, VLCAD_flag low
ATPB_HUMAN [45]	57 kDa	0.0037	EV_flag low, MMAB_flag high, VLCAD_flag low
ODPB_HUMAN	39 kDa	0.0039	EV_flag low, MMAB_flag high, VLCAD_flag low
C1TM_HUMAN	106 kDa	0.0039	EV_flag low, MMAB_flag high, VLCAD_flag low
HCDH_HUMAN	34 kDa	0.0039	EV_flag low, MMAB_flag high, VLCAD_flag low
ALDH2_HUMAN	56 kDa	0.0039	EV_flag low, MMAB_flag high, VLCAD_flag low
EFTU_HUMAN	50 kDa	0.0044	EV_flag low, MMAB_flag high, VLCAD_flag low
MDHM_HUMAN	36 kDa	0.0053	EV_flag low, MMAB_flag high, VLCAD_flag low
AATM_HUMAN	48 kDa	0.0076	EV_flag low, MMAB_flag high, VLCAD_flag low
ECHB_HUMAN	51 kDa	0.0076	EV_flag low, MMAB_flag high, VLCAD_flag low
TRAP1_HUMAN [2]	80 kDa	0.0085	EV_flag low, MMAB_flag high, VLCAD_flag low
TRAP1_HUMAN	80 kDa	0.0078	EV_flag low, MMAB_flag high, VLCAD_flag low
THIL_HUMAN	45 kDa	0.013	EV_flag low, MMAB_flag high, VLCAD_flag low
CISY_HUMAN	52 kDa	0.014	EV_flag low, MMAB_flag high, VLCAD_flag low
SSBP_HUMAN	17 kDa	0.014	EV_flag low, MMAB_flag high, VLCAD_flag low
P5CS_HUMAN	87 kDa	0.02	EV_flag low, MMAB_flag high, VLCAD_flag low
RT23_HUMAN	22 kDa	0.022	EV_flag low, MMAB_flag high, VLCAD_flag low
GLYM_HUMAN [2]	56 kDa	0.025	EV_flag low, MMAB_flag high, VLCAD_flag low
QCR1_HUMAN	53 kDa	0.027	EV_flag low, MMAB_flag high, VLCAD_flag low
ATPA_HUMAN [2]	60 kDa	0.031	EV_flag low, MMAB_flag high, VLCAD_flag low
ATPG_HUMAN	33 kDa	0.031	EV_flag low, MMAB_flag high, VLCAD_flag low
GRP75_HUMAN	74 kDa	0.033	EV_flag low, MMAB_flag high, VLCAD_flag low
CH60_HUMAN [2]	61 kDa	0.037	EV_flag low, MMAB_flag high, VLCAD_flag low
**MCEE-pulldown**			
THIL_HUMAN	45 kDa	0.00048	EV_flag low, MCEE_flag high, VLCAD_flag low
MCEE_HUMAN	19 kDa	0.0012	EV_flag low, MCEE_flag high, VLCAD_flag low
ODO2_HUMAN	49 kDa	0.0012	EV_flag low, MCEE_flag high, VLCAD_flag low
CALX_HUMAN	68 kDa	0.0012	EV_flag low, MCEE_flag high, VLCAD_flag low
ODPB_HUMAN	39 kDa	0.0039	EV_flag low, MCEE_flag high, VLCAD_flag low
THIM_HUMAN	42 kDa	0.0039	EV_flag low, MCEE_flag high, VLCAD_flag low
HCD2_HUMAN	27 kDa	0.0052	EV_flag low, MCEE_flag high, VLCAD_flag low

GRPE1_HUMAN (+1)	24 kDa	0.0052	EV_flag low, MCEE_flag high, VLCAD_flag low
ECHB_HUMAN	51 kDa	0.0052	EV_flag low, MCEE_flag high, VLCAD_flag low
ACADM_HUMAN (+1)	47 kDa	0.0076	EV_flag low, MCEE_flag high, VLCAD_flag low
PGAM5_HUMAN	32 kDa	0.008	EV_flag low, MCEE_flag high, VLCAD_flag low
RPN1_HUMAN	69 kDa	0.008	EV_flag low, MCEE_flag high, VLCAD_flag low
ALDH2_HUMAN	56 kDa	0.008	EV_flag low, MCEE_flag high, VLCAD_flag low
GLYM_HUMAN [2]	56 kDa	0.0088	EV_flag low, MCEE_flag high, VLCAD_flag low
ETFB_HUMAN	28 kDa	0.0091	EV_flag low, MCEE_flag high, VLCAD_flag low
MDHM_HUMAN	36 kDa	0.011	EV_flag low, MCEE_flag high, VLCAD_flag low
ATPG_HUMAN	33 kDa	0.011	EV_flag low, MCEE_flag high, VLCAD_flag low
EFTU_HUMAN	50 kDa	0.013	EV_flag low, MCEE_flag high, VLCAD_flag low
CH10_BOVIN (+1)	11 kDa	0.015	EV_flag low, MCEE_flag high, VLCAD_flag low
AATM_HUMAN	48 kDa	0.016	EV_flag low, MCEE_flag high, VLCAD_flag low
DHE3_HUMAN [2]	61 kDa	0.019	EV_flag low, MCEE_flag high, VLCAD_flag low
CH60_HUMAN [2]	61 kDa	0.019	EV_flag low, MCEE_flag high, VLCAD_flag low
ATD3A_HUMAN [2]	71 kDa	0.021	EV_flag low, MCEE_flag high, VLCAD_flag low
SSBP_HUMAN	17 kDa	0.021	EV_flag low, MCEE_flag high, VLCAD_flag low
ATPA_HUMAN [2]	60 kDa	0.022	EV_flag low, MCEE_flag high, VLCAD_flag low
ATPO_HUMAN	23 kDa	0.025	EV_flag low, MCEE_flag high, VLCAD_flag low
ATPB_HUMAN [45]	57 kDa	0.026	EV_flag low, MCEE_flag high, VLCAD_flag low
RL34_HUMAN	13 kDa	0.027	EV_flag low, MCEE_flag high, VLCAD_flag low
TRAP1_HUMAN [2]	80 kDa	0.028	EV_flag low, MCEE_flag high, VLCAD_flag low
TRAP1_HUMAN	80 kDa	0.028	EV_flag low, MCEE_flag high, VLCAD_flag low
RT23_HUMAN	22 kDa	0.031	EV_flag low, MCEE_flag high, VLCAD_flag low
SDHA_HUMAN (+1)	73 kDa	0.031	EV_flag low, MCEE_flag high, VLCAD_flag low
SSDH_HUMAN	57 kDa	0.031	EV_flag low, MCEE_flag high, VLCAD_flag low
ODP2_HUMAN	69 kDa	0.034	EV_flag low, MCEE_flag high, VLCAD_flag low
OAT_HUMAN	49 kDa	0.034	EV_flag low, MCEE_flag high, VLCAD_flag low
ATP5H_HUMAN	18 kDa	0.034	EV_flag low, MCEE_flag high, VLCAD_flag low
IPYR2_HUMAN	38 kDa	0.034	EV_flag low, MCEE_flag high, VLCAD_flag low
CISY_HUMAN	52 kDa	0.05	EV_flag low, MCEE_flag high, VLCAD_flag low
**MMAA-pulldown**			
MMAA_HUMAN	47 kDa	0.00028	EV_flag low, MMAA_flag high, VLCAD_flag low
MUTA_HUMAN [3]	83 kDa	0.0032	EV_flag low, MMAA_flag high, VLCAD_flag low
ETFB_HUMAN	28 kDa	0.027	EV_flag low, MMAA_flag high, VLCAD_flag low
RT23_HUMAN	22 kDa	0.027	EV_flag low, MMAA_flag high, VLCAD_flag low

Supplementary Document 1. Questionnaire to obtain the phenotype data.

Methylmalonic aciduria patient questionnaire

Basic patient information

- Name: ....
- First name: ....
- Date of birth: ....
- Birth weight: ....
- Gestational age at birth: ....
- APGAR score: ....
- Pregnancy (abnormalities of the fetus, symptoms of the mother): ....
- Consanguinity of the parents (y/n, relation of parents): ....
- Nationality: ....
- Siblings (age, symptoms, medical history): ....
- Social situation: ....
- Profession of parents: ....

Diagnosis

- Date: ....
- Enzymatic results: ....
- Genetic results: ....
- First presenting symptoms: ....
- All clinical findings at diagnosis: ....
  ....
- Onset of symptoms (age): ....
- Hospitalizations (date, place): ....
- Weight: ....
- Length: ....
- Head circumference: ....

Lab findings at diagnosis (provide units)

- pH: ....
- BE: ....
- NH3: ....
- Blood count: ....
- Free carnitine (plasma): ....
- Total carnitine (plasma): ....
- Glucose (plasma): ....
- Vitamin B12 (plasma): ....
- Homocysteine (plasma): ....
- Methylmalonic acid (plasma): ....
- Ketone bodies (urine): ....
- Methylmalonic acid/creatinine ratio (urine): ....
- 2-Methylcitrate/creatinine ratio (urine): ....
- Free carnitine/creatinine ratio (urine): ....
- Total carnitine/creatinine ratio (urine): ....
- Odd-numbered long-chain fatty acids (red blood cells): ....

Treatment of acute episodes (including initial presentation, please indicate)

- Glucose IV (y/n, duration, amount): ....
- Insulin (y/n, duration, amount): ....
- Bicarbonate IV (y/n, duration, amount): ....
- Peritoneal dialysis (y/n, duration): ....
- Exchange transfusion (y/n, duration): ....
- Vitamin B12 (OH-Cbl, CN-Cbl; dose; route): ....
- Responsive to Vitamin B12 treatment (y/n, how assessed): ....
- Other: ....

Diet/treatment (at discharge after initial presentation)

- Natural protein (g/kg/day): ....
- Synthetic protein (g/kg/day) (which amino acid mixtures): ....
- Antibiotics for gut sterilization (name of antibiotics): ....
- Carnitine (mg/kg/day): ....
- Other: ....
- Response to treatment (clinical course, biochemical changes): ....
- Further hospitalizations and their course (see above to include presenting symptoms and biochemistry, and treatment modalities): ....
....
....
- General progress of the patient after initial presentation (e.g. complications): ....
....
....
- Psychomotor development after initial presentation (including milestones): ....
....
....

Details of long-term treatment

- Number of meals per day: ....
- Calories/kg/day: ....
- Tube feeding (y/n; intermittent/day/night): ....
- Natural protein intake (g/kg/day): ....
- Synthetic protein intake (g/kg/day; details of amino acid mixture): ....
- Vitamin B12 treatment (OH-Cbl, CN-Cbl; dose; frequency; route): ....
- Carnitine (mg/kg/day): ....
- Other medication: ....
- Feeding problems (y/n): ....
- Compliance with treatment: ....

Laboratory assessments during regular follow-up (provide units)

- Metabolic acidosis (pH): ....
- Base excess: ....
- Methylmalonic acid (plasma): ....
- Methylmalonic acid (CSF): ....
- Methylmalonic acid/creat. ratio (urine): ....
- Odd-numbered long-chain fatty acids (red blood cells): ....

Long-term clinical follow-up

- Weight (with age at measurement): ....
- Length (with age at measurement): ....
- Head circumference (with age at measurement): ....
- Psychomotor development (milestones): ....
- Intellectual quotient, developmental quotient (with age at assessment): ....
- Schooling (grade, special schooling): ....
- Neurological abnormalities (muscle tone, epilepsy, hearing, vision problems, movement disorder): ....
....
....
- EEG (with details of findings): ....
....
....
- MRI / CT (with details of findings): ....
....
....
- Impaired kidney function (y/n; how assessed): ....
- GFR (e.g. creatinine clearance): ....
- Kidney ultrasound (with details of report): ....
....
....
- Uric acid (provide units): ....
- Creatinine (provide units): ....
- Blood pressure (provide units): ....
- Hematologic abnormalities: ....
- Complications, additional clinical problems (e.g. cardiomyopathy, signs of metabolic stroke, pancreatitis, skin abnormalities, other): ....
....
....

Deceased (y/n; age; cause): ....

Contact details of treating physician (Address, phone number, email): ....
....
....

Date: ....

Signature (treating physician): ....
