# Supplementary material for: Integrated multi-omics reveals anaplerotic rewiring in methylmalonyl-CoA mutase deficiency
Source: Nat Metab. 2023 Jan 26;5(1):80–95. doi: 10.1038/s42255-022-00720-8 (PMC9886552; doi:10.1038/s42255-022-00720-8)
Supplement: Supplementary file 2 — Reporting Summary [file 42255_2022_720_MOESM2_ESM.pdf]

nature portfolio

Corresponding author(s): Sean Froese

Last updated by author(s): 24 Nov, 2022

Reporting Summary

Nature Portfolio wishes to improve the reproducibility of the work that we publish. This form provides structure for consistency and transparency in reporting. For further information on Nature Portfolio policies, see our Editorial Policies and the Editorial Policy Checklist.

Statistics

For all statistical analyses, confirm that the following items are present in the figure legend, table legend, main text, or Methods section.

n/a	Confirmed	
☐	☒	The exact sample size (n) for each experimental group/condition, given as a discrete number and unit of measurement
☐	☒	A statement on whether measurements were taken from distinct samples or whether the same sample was measured repeatedly
☐	☒	The statistical test(s) used AND whether they are one- or two-sided<br>*Only common tests should be described solely by name; describe more complex techniques in the Methods section.*
☐	☒	A description of all covariates tested
☐	☒	A description of any assumptions or corrections, such as tests of normality and adjustment for multiple comparisons
☐	☒	A full description of the statistical parameters including central tendency (e.g. means) or other basic estimates (e.g. regression coefficient) AND variation (e.g. standard deviation) or associated estimates of uncertainty (e.g. confidence intervals)
☐	☒	For null hypothesis testing, the test statistic (e.g. F, t, r) with confidence intervals, effect sizes, degrees of freedom and P value noted<br>*Give P values as exact values whenever suitable.*
☒	☐	For Bayesian analysis, information on the choice of priors and Markov chain Monte Carlo settings
☒	☐	For hierarchical and complex designs, identification of the appropriate level for tests and full reporting of outcomes
☐	☒	Estimates of effect sizes (e.g. Cohen's d, Pearson's r), indicating how they were calculated

Our web collection on statistics for biologists contains articles on many of the points above.

Software and code

Policy information about availability of computer code

Data collection	Genomic data was collected using the NovaSeq Control Software v1.6 software on the NovaSeq 6000 sequencer.<br>RNA-seq data was collected using the HCS 3.4.0 Software v3.4.0 software on the Illumina HiSeq 4000.<br>DIA proteomics data was collected using the Q Exactive HF Tune v2.4 software on a Q Exactive HF mass spectrometer.<br>Metabolomics information in primary fibroblasts was collected using the Agilent MassHunter Software B.07.00 on the Agilent 6550 QTOF instrument.
Data analysis	Initial data analysis of the DIA proteomics data was done using Spectronaut v12 (Biognosys).<br>Data analysis was performed using R version 4.1.0, including packages MOFA 1.3.1 22, MASS 7.3-54 56, fgsea 1.18.0 57, circlize 0.4.13 58.

For manuscripts utilizing custom algorithms or software that are central to the research but not yet described in published literature, software must be made available to editors and reviewers. We strongly encourage code deposition in a community repository (e.g. GitHub). See the Nature Portfolio guidelines for submitting code & software for further information.

Data

Policy information about availability of data

All manuscripts must include a data availability statement. This statement should provide the following information, where applicable:

- Accession codes, unique identifiers, or web links for publicly available datasets
- A description of any restrictions on data availability
- For clinical datasets or third party data, please ensure that the statement adheres to our policy

Access to the raw genomic and transcriptomic data is restricted due to ethical concerns. Data can be made available upon reasonable request to D.S.F. within a timeframe of 3 months following an established data transfer and use agreement and ethical approval. The mass spectrometry proteomics data (.raw files) have

been deposited to the ProteomeXchange Consortium (http://proteomecentral.proteomexchange.org) via the MassIVE partner repository (https://massive.ucsd.edu) with the dataset identifiers MSV000090551 and PXD038225. Metabolomics mass spectrometry raw data for human fibroblast measurements have been uploaded to the MassIVE data repository (https://massive.ucsd.edu) with the dataset identifier MSV000089082. IP-MS raw files have been deposited to the ProteomeXchange Consortium via the MassIVE partner repository (https://massive.ucsd.edu) with the dataset identifier MSV000088791.

Field-specific reporting

Please select the one below that is the best fit for your research. If you are not sure, read the appropriate sections before making your selection.

[x] Life sciences [ ] Behavioural & social sciences [ ] Ecological, evolutionary & environmental sciences

For a reference copy of the document with all sections, see nature.com/documents/nr-reporting-summary-flat.pdf

Life sciences study design

All studies must disclose on these points even when the disclosure is negative.

Sample size	No sample-size calculation was performed. Instead, all samples available with suspicion of the rare metabolic disease methylmalonic aciduria at our diagnostic center were used.
Data exclusions	In the RNA-seq dataset, nine samples were excluded as the initial RNA extraction did not yield sufficient material for further processing. This exclusion criterion was defined prior to the analysis. Details about which samples are affected are noted in the Methods section of the manuscript. No other data obtained during the course of the study were excluded from the analysis.
Replication	Whenever possible replicative measurements were taken at the biological as well as the technical level. For CRISPR/Cas9 engineered cells clonal replicates were generated to account for clonal variability. Details of replicates are described in the Methods section and the figure legends of the manuscript. All experiments were successfully replicated.
Randomization	For WGS, RNA-seq, proteomics, and metabolomics samples were randomized within blocks (for details see Methods section of the manuscript). For data interpretation samples were grouped according to their genotypes.
Blinding	For WGS, RNA-seq, proteomics, and metabolomics experimenters were blinded with regards to the experimental group of the samples.

Reporting for specific materials, systems and methods

We require information from authors about some types of materials, experimental systems and methods used in many studies. Here, indicate whether each material, system or method listed is relevant to your study. If you are not sure if a list item applies to your research, read the appropriate section before selecting a response.

Materials & experimental systems

n/a	Involved in the study	
[ ]	[x]	Antibodies
[ ]	[x]	Eukaryotic cell lines
[x]	[ ]	Palaeontology and archaeology
[ ]	[x]	Animals and other organisms
[ ]	[x]	Human research participants
[x]	[ ]	Clinical data
[x]	[ ]	Dual use research of concern

Methods

n/a	Involved in the study	
[x]	[ ]	ChIP-seq
[x]	[ ]	Flow cytometry
[x]	[ ]	MRI-based neuroimaging

Antibodies

Antibodies used	Primary antibodies:<br>MMUT (Abcam, ab67869, 1:1000, host: mouse), OGDH (Atlas antibodies, HPA020347, 1:500, host: rabbit), GLUD (Abcam, ab166618, 1:2000, host: rabbit), Beta-actin (Sigma, A1978, 1:5000, host: mouse)<br><br>Secondary antibodies:<br>Anti-rabbit HRP (Santa Cruz, sc-2357, 1:5000, host: mouse), Anti-mouse HRP (Santa Cruz, sc-516102, 1:5000, host: goat).
Validation	Validation of commercially available primary antibodies can be found on the vendor's website (accessed 20 April 2022):<br>- MMUT (Abcam, ab67869): https://www.abcam.com/methylmalonyl-coenzyme-a-mutase-antibody-ab67869.html<br>- OGDH (Atlas antibodies, HPA020347): https://www.atlasantibodies.com/products/antibodies/primary-antibodies/triple-a-polyclonals/ogdh-antibody-hpa020347/<br>- GLUD (Abcam, ab166618): https://www.abcam.com/glud1--glud2-antibody-epr11369b-ab166618.html<br>- Beta-actin (Sigma, A1978): https://www.sigmaaldrich.com/CH/de/product/sigma/a1978

Eukaryotic cell lines

Policy information about cell lines

Cell line source(s)	Primary fibroblast cells: Tissue bio bank University Children's Hospital Zurich<br>HEK293T cells: ATCC CRL-3216 (https://www.atcc.org/products/crl-3216, accessed 21 April 2022)
Authentication	Cell lines were not authenticated.
Mycoplasma contamination	All cell lines were tested periodically for mycoplasma contamination with negative results.
Commonly misidentified lines<br>(See ICLAC register)	No misidentified cell lines were used.

Animals and other organisms

Policy information about studies involving animals; ARRIVE guidelines recommended for reporting animal research

Laboratory animals	Mmut-ko/ki control Mmut-ki/wt mice (Mus musculus) were bred by crossing Mmut-ko/wt female and Mmut-ki/ki male mice. The background was C57BL/6J. Experimental mice were female. Aged 58-63 days.
Wild animals	Study did not involve wild animals.
Field-collected samples	Study did not involve wild animals.
Ethics oversight	All animal experiments were approved by the Cantonal Veterinary Office Zurich (license number 202/2014).

Note that full information on the approval of the study protocol must also be provided in the manuscript.

Human research participants

Policy information about studies involving human research participants

Population characteristics	The presented study involved a cohort of individuals suspected to be affected by the rare metabolic disease methylmalonic aciduria. Due to the rarity of the condition, heterogeneity of the study population has to be taken into account to achieve relevant samples sizes. Most findings of this study are based on the multi-omics datasets obtained by analysing primary fibroblast cells derived from the study cohort. As all these cell lines were cultivated under standard conditions, cohort heterogeneity is negligable for these results.<br>The genotypes of the individuals in our cohort were investigated as part of this study. Please refer to the first section of the results.<br>Regarding the phenotypic traits of the individuals in our cohort, we provide a detailed overview of their main clinical and biochemical features. Please refer to Source data table 1.
Recruitment	The cohort was compiled based on the availability of samples derived from individuals with suspicion of the rare metabolic disease methymalonic aciduria at our diagnostic reference center. Unaffected inviduals constituted unaffected siblings and inviduals suspected with disorders unrelated to the current study where no defect was detected.
Ethics oversight	Usage of primary fibroblast cells and the associated phenotypic data of individuals was approved by the Ethics committee of the Canton of Zurich, Switzerland (KEK-2014-0211, amendment: PB_2020-00053).

Note that full information on the approval of the study protocol must also be provided in the manuscript.
