# Supplementary figures and images for: Integrated multi-omics reveals anaplerotic rewiring in methylmalonyl-CoA mutase deficiency
Source: Nat Metab. 2023 Jan 26;5(1):80–95. doi: 10.1038/s42255-022-00720-8 (PMC9886552; doi:10.1038/s42255-022-00720-8)

Figure 6e  
Unmodified blots

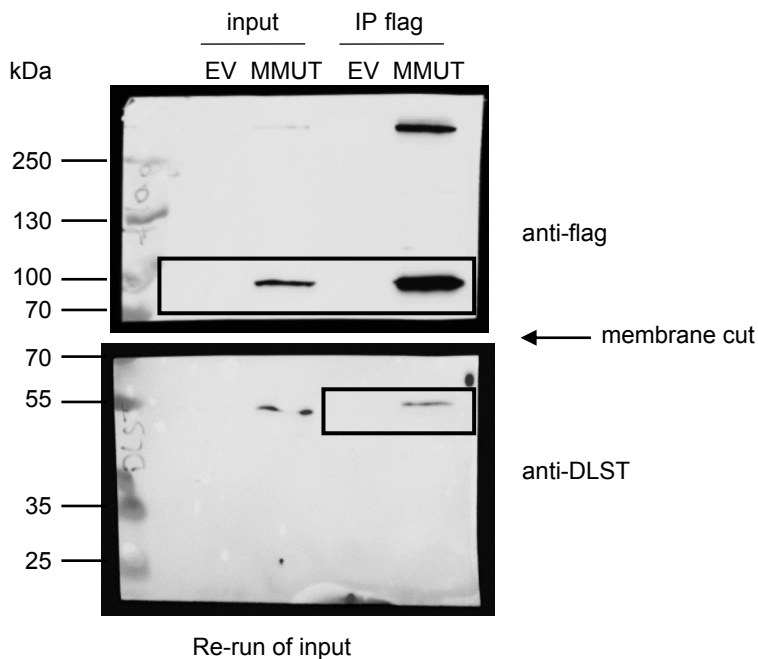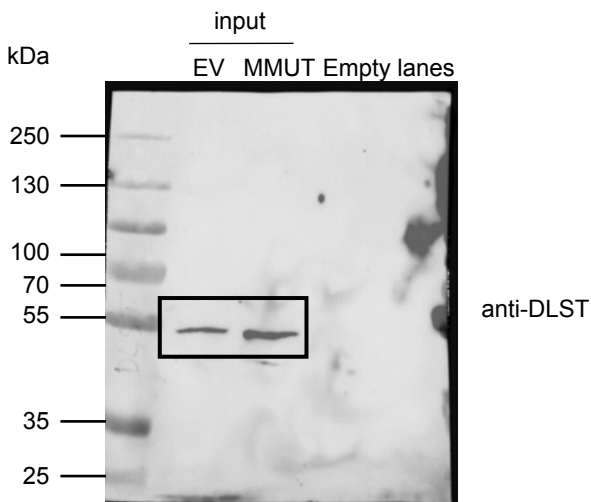

Supplement: Source Data Fig. 6 — Unprocessed Western blots. [file 42255_2022_720_MOESM8_ESM.pdf]

# HEK293 wt

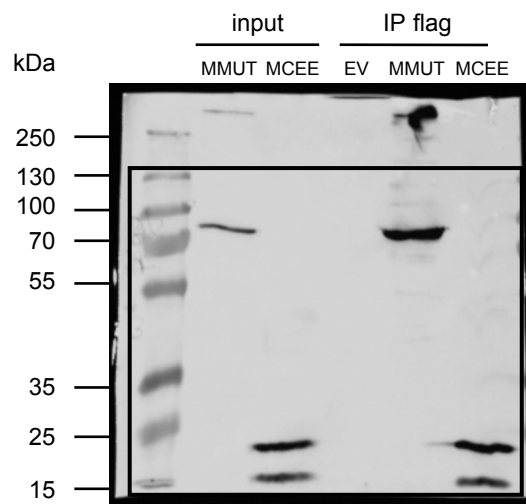

# HEK293 MUT KO

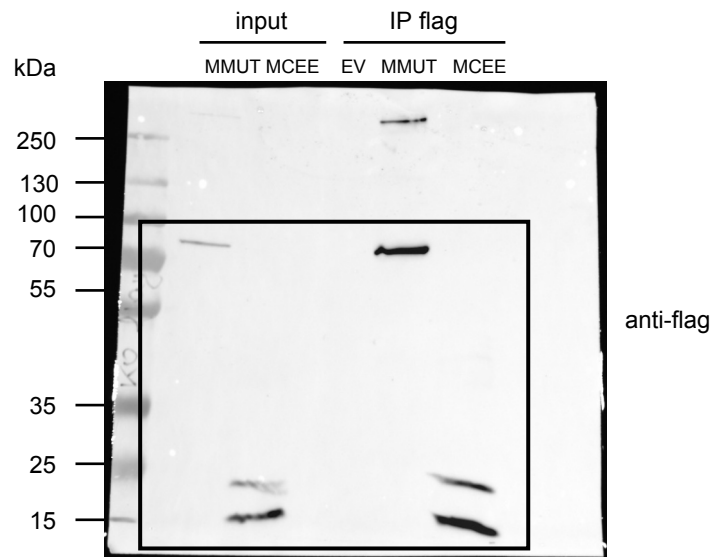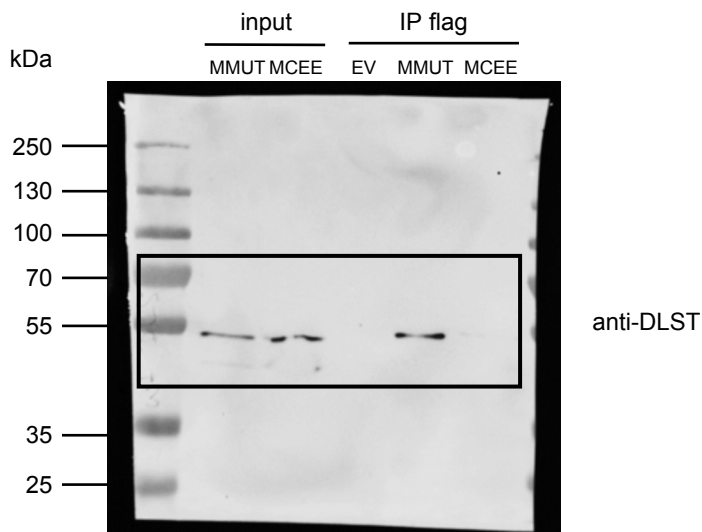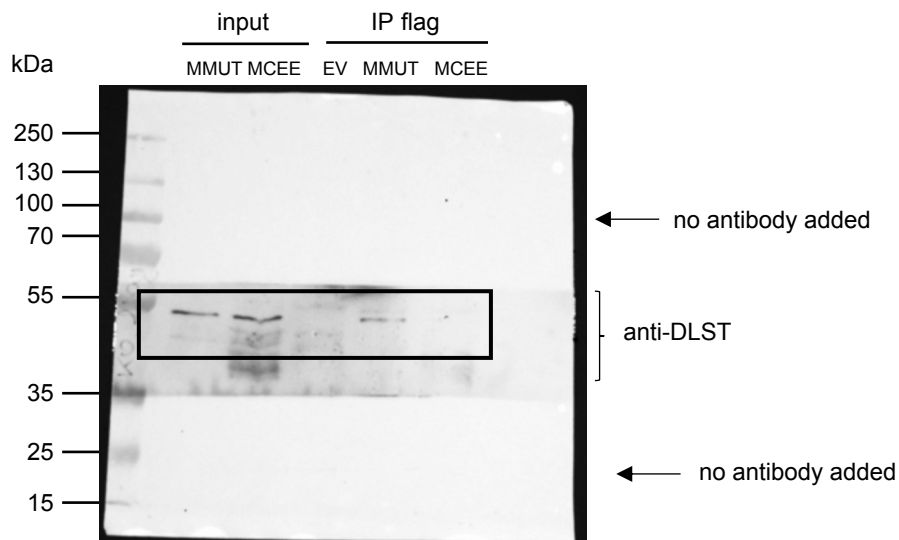

Supplement: Source Data Extended Data Fig. 10 — Unprocessed Western blots. [file 42255_2022_720_MOESM10_ESM.pdf]
