# Supplementary material for: Integrated multi-omics reveals anaplerotic rewiring in methylmalonyl-CoA mutase deficiency
Source: Nat Metab. 2023 Jan 26;5(1):80–95. doi: 10.1038/s42255-022-00720-8 (PMC9886552; doi:10.1038/s42255-022-00720-8)

Extended Data Figure 9b (left panel)
Unmodified blots

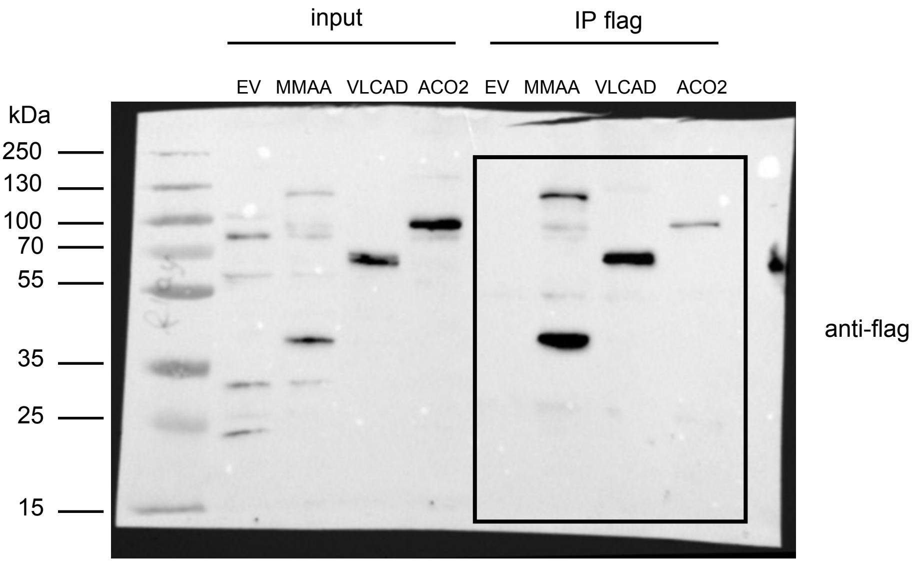

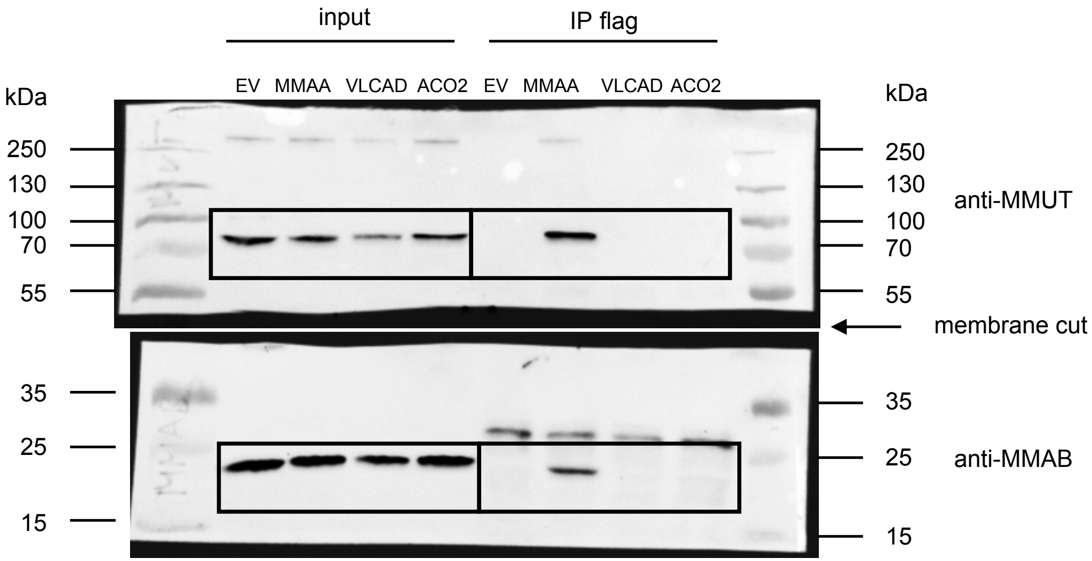

Extended Data Figure 9b (right panel, part 1)
Unmodified blots

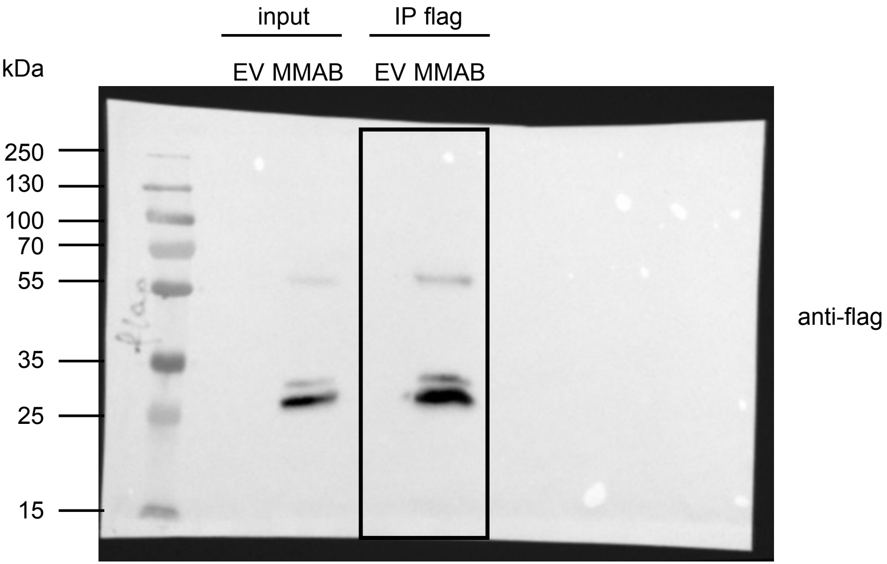

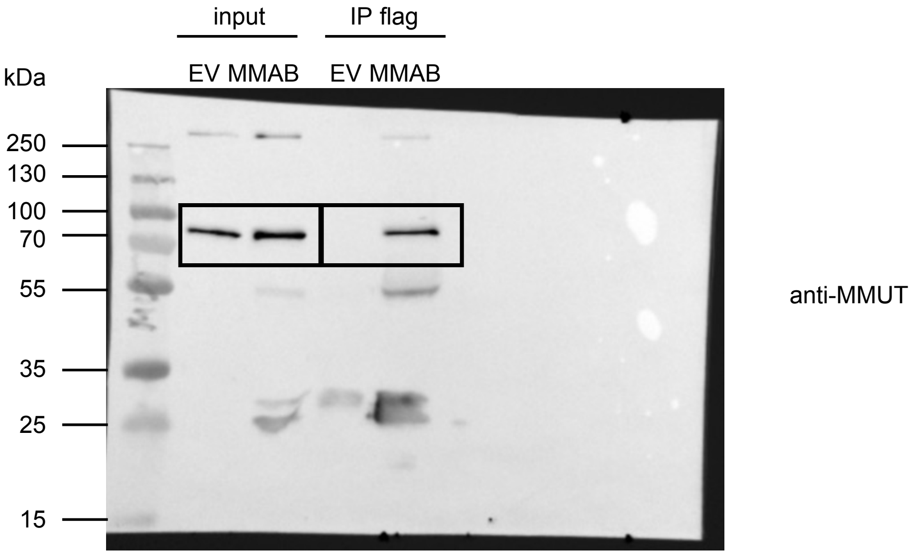

Extended Data Figure 9b (right panel part 2)
Unmodified blots

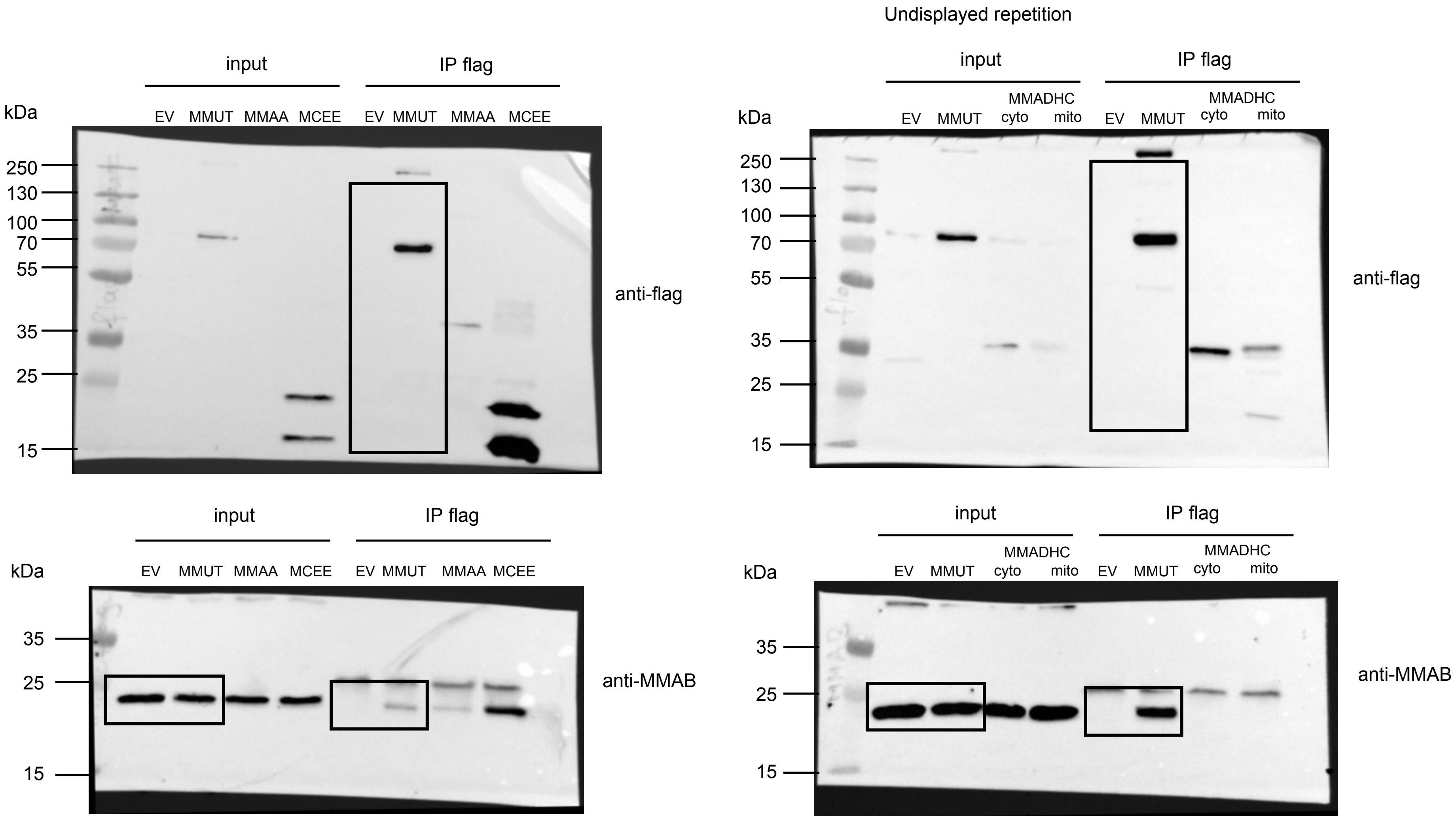

Extended Data Figure 9b (right panel part 3)
Unmodified blots

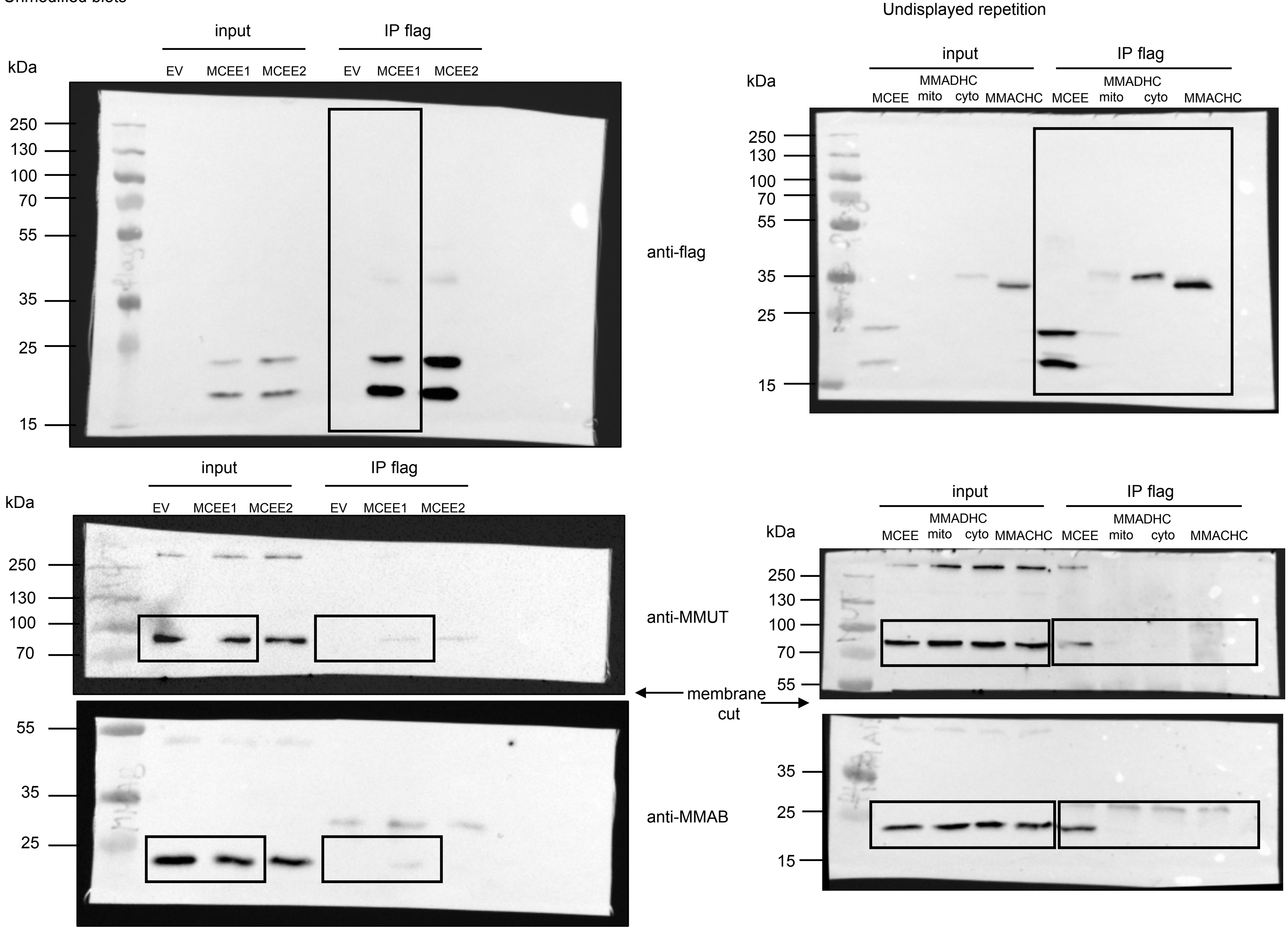

Extended Data Figure 9c
Unmodified blots

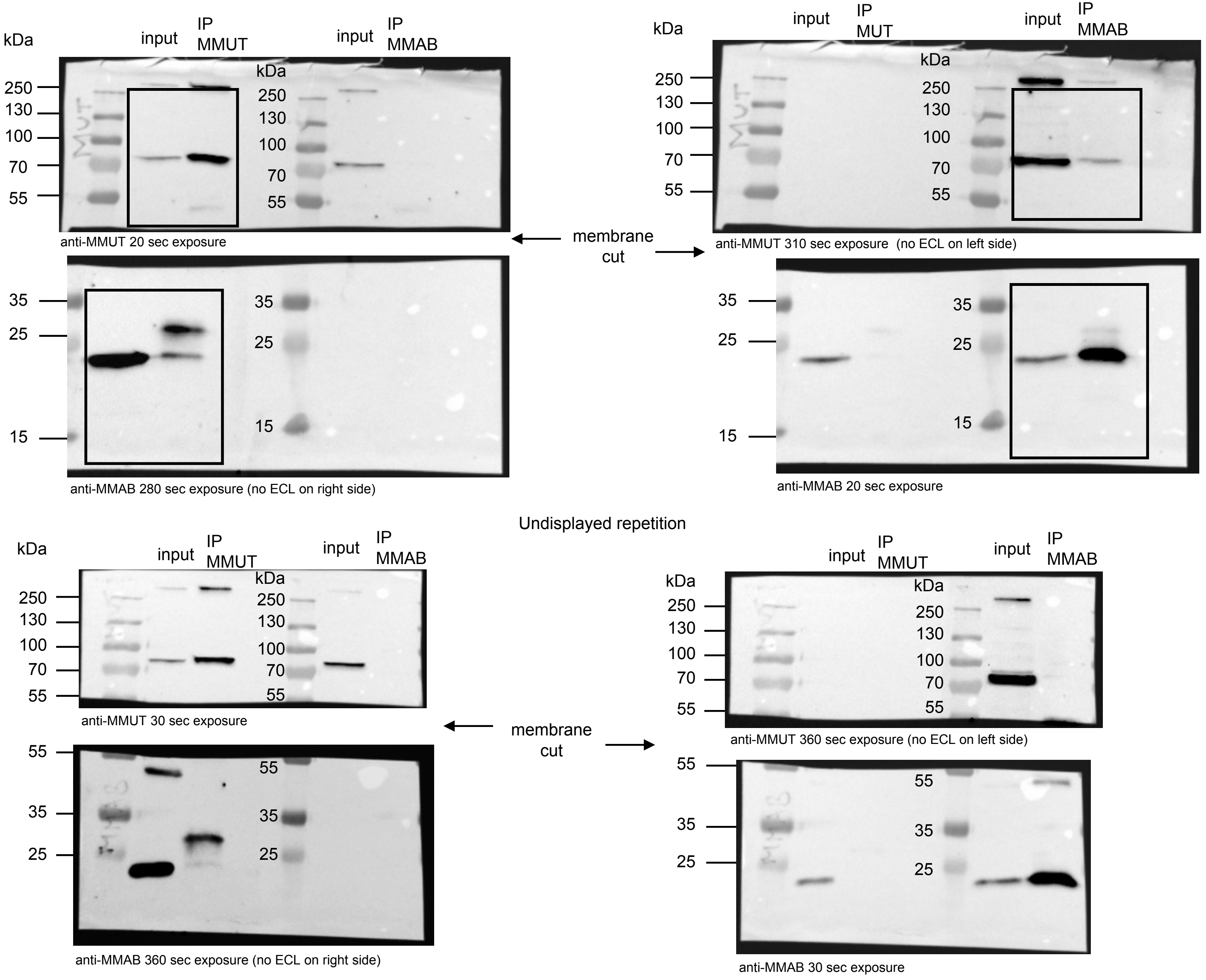

Supplement: Source Data Extended Data Fig. 9 — Unprocessed Western blots. [file 42255_2022_720_MOESM9_ESM.pdf]
